# Torsional behaviour of supercoiled DNA regulates recognition of architectural protein Fis on minicircle DNA

**DOI:** 10.1093/nar/gkac522

**Published:** 2022-06-24

**Authors:** Anupam Mondal, Sangeeta, Arnab Bhattacherjee

**Affiliations:** School of Computational and Integrative Sciences, Jawaharlal Nehru University, New Delhi 110067, India; School of Computational and Integrative Sciences, Jawaharlal Nehru University, New Delhi 110067, India; School of Computational and Integrative Sciences, Jawaharlal Nehru University, New Delhi 110067, India

## Abstract

Three-dimensional genome organization is indispensable for regulating its functions. Experimental techniques based on the proximity ligation principle have identified spatially segregated domains at a sub-megabase level. The transcriptional activity of these domains is strongly coupled with the supercoiled DNA topology. The underlying molecular principle however remains unknown. By developing a computational framework, we investigate the differential kinetics of an architectural protein Fis on supercoiled DNA. We find that DNA supercoiling favours formation of juxtaposition sites where proteins can perform intersegmental transfer between the spatially close sites. The juxtaposition sites in positively supercoiled DNA are torsionally pinned that results in a slow protein diffusion on it, whereas the torsional behaviour of negatively supercoiled DNA ensures rapid protein-DNA communication and overall fast recognition of the cognate site by proteins. The result is robust for other proteins irrespective of their molecular features. Furthermore, our study suggests that variation in protein diffusivity on different supercoiled DNA also influences the shape of the latter. By unravelling the underlying molecular picture, our results unravel a plausible link between the degree of DNA supercoiling and the regulation of gene transcription that significantly advances the current understanding of the function and organization of chromatin inside cells.

## INTRODUCTION

Three dimensional (3D) genome organization tightly regulates gene expression during development and cell differentiation. A fundamental component of the organization is DNA supercoiling, which is intrinsic to genome architecture (1–4) due to topological constraints imposed by tethering of DNA ends inside the bacterial cell membrane or anchoring to the chromosome scaffold in eukaryotes (5–8). The topology of supercoiled DNA is regulated by the interplay of two parameters, namely, DNA twisting (*Tw*) and bending deformations (*Wr*). The relation *Lk* = *Tw* + *Wr* connects twist (*Tw*), defined as the total number of helical turns and writhe (*Wr*) of topologically constrained DNA that indicates the bending of DNA helical axis around itself in three dimensions. *Lk* defines the topologically invariant linking number. Any change in *Lk* from its relaxed value (*Lk*_0_) causes supercoiling in DNA. The corresponding change in linking number Δ*Lk* = *Lk* − *Lk*_0_ = Δ*Tw* + Δ*Wr* can be described in terms of deviations in the twist and writhe from their equilibrium values. Changes in twist value can create DNA bubble formation (9) or cruciform DNA (10) and modulate the binding of proteins (11,12), whereas, alteration of writhe results in the appearance of plectoneme structures (13–15). *In vivo*, the supercoiled DNA states are known to regulate gene expressions (16), enzyme binding (17), and genome organization (18). Indeed, the bacterial chromatin organizes to independent domains (19) where highly transcribed genes are found to be positioned in domains with underwound (negatively supercoiled) DNA conformations whereas the domains containing inactive genes are generally overwound (20,21). Investigation along the line confirms that around 200 genes show repressed expression in *Escherichia coli*, whereas 106 genes show increased expression with the change in DNA supercoiling, comprising as much as 7% of all genes in *E. coli* responsive to the alterations in DNA supercoiling (22–25). Furthermore, transcription of a gene itself can alter the local supercoiling of DNA, commonly builds up positive supercoiling in DNA segments. Upon accumulation, the resulting torsional strain acts as a cellular switch to halt DNA transcription that resumes upon gyrase binding, explaining the origin of stochastic bursts observed in the transcription of highly expressed genes in bacteria (26). Unlike bacterial chromatin, in eukaryotes, DNA is wrapped as a toroid around a nucleosome core particle, where the handedness of the DNA wrapping is intrinsically ambidextrous and relies on the supercoiling state of the DNA (27). During transcription, RNA polymerase (RNAp) progresses and generates positive DNA supercoils (28) that destabilize nucleosomes and promote transient access of the nucleosomal DNA, which are otherwise occluded due to histone occupancy. Negative supercoils that form behind RNAp promote nucleosome assembly (28). Despite the differences between bacteria and eukaryotes in their mode of packaging DNA supercoils, a common trait of genetic regulation that positive supercoiling is associated with transcriptional silencing and negative supercoiling is linked with the acquisition of transcriptional competence, can be observed in both (29).

Notwithstanding the wealth of information supporting the connection between DNA supercoiling and transcription, the underlying mechanism of how DNA supercoiling may regulate the level of gene transcription is largely unknown. Primarily, it is because of the experimental difficulties associated with measuring supercoiling in active DNA (30) and lack of availability of supercoiled substrates used in quantitative binding assays of DNA binding proteins (DBPs) (31). Here we address the issue by considering recognition of the target site on a nucleosome free DNA minicircle by a DBP as an elementary step to trigger transcription of gene and probing how the DNA supercoiling may influence this step. A number of studies previously attempted to unravel the molecular mechanism of searching the target DNA sites by DBPs using linear stretches of DNA as scaffold (32–37). The emerged generalized picture suggests that a facilitated diffusion process accelerates the target search of DBPs by lowering the dimension of their search space (36,38–41). The protein nonspecifically associates with the linear DNA segment at any arbitrary site and performs a combination of 3D and 1D search dynamics instead of performing a pure 3D diffusion. The advantage of using a combination of 3D and 1D search modes is that the protein in 3D can diffuse fast to reach different segments of the DNA, while in the 1D mode of translocation, the protein can read out the DNA bases closely to specifically recognize the target DNA sequences. An exclusive 3D or 1D diffusion allows the protein to diffuse either extremely fast without specifically scanning the DNA sequences or scanning the DNA sequences precisely but at the cost of reduced search speed. In both ways, the recognition of the target DNA site is much slower which explains why a trade-off between 3D and 1D search modes facilitates the overall search process. The model while highlights the role of protein dynamics in searching DNA target sites, it severely underrates the role of conformational heterogeneity and dynamics of DNA (31), which we previously confirmed as significant as protein dynamics in regulating the protein−DNA recognition (42,43).

We, therefore develop a computational framework that can capture the conformational heterogeneity of supercoiled DNA minicircle observed in nature. We verify our results against the supercoiled topology predicted by cryo-ET experiments (44) as well as results obtained from an all-atom simulation (44). The model is then suitably amalgamated with a coarse-grained description of an architectural protein Fis to probe the role of DNA supercoiling in modulating the protein dynamics and its target search efficiency on supercoiled DNA. We find a strong relationship between the torsional behaviour of supercoiled DNA and the efficiency of Fis in recognizing their target DNA sites. By examining the footmark of the protein during our simulations, we show that the positively supercoiled DNA is torsionally pinned to result in a slow Fis diffusion. While this undermines the recognition of target DNA sites by Fis, the slow Fis dynamics instigates alteration in spatial architecture of the positively supercoiled DNA conformations. The torsional behaviour of negatively supercoiled plectonemes however, is found to speed up the Fis diffusion that promotes rapid communications of the protein with sequentially distal DNA segments and helps in recognizing the cognate DNA site. Our results capture the underlying mechanism that correlates DNA supercoiling with Fis diffusivity, which we further confirmed by comparing the molecular simulation results with a previously developed statistical mechanical model by us. An excellent agreement between the two indicates that the proposed computational framework provides a comprehensive understanding of recognition of target sites by Fis on the topologically constrained genomic DNA that are otherwise difficult to probe. Importantly, our findings are independent of the interacting protein, although the extent of differences in protein diffusivity and its influence in altering the DNA architecture may vary. The results are therefore, a significant advancement in linking the degree of DNA supercoiling with the regulation of gene transcription and chromatin organization inside cells with a detailed molecular picture.

## MATERIALS AND METHODS

### Protein model

The dynamics of protein diffusion on DNA was studied using a coarse-grained model. The resolution of the model is such that it enable us to investigate the large-scale biomolecular processes at long time scales that are inaccessible to all-atom model. In this study, the structure of a protein molecule is denoted by a coarse-grained *C*_α_ model, where each amino acid is represented by a single bead centered on its α-carbon (*C*_α_) position (45). The energetics of the protein molecule is described by a native topology based model that uses a Lennard–Jones potential to incorporate the native contacts found in the crystal structure (46). Such structure based potential represents a funnel-like energy landscape for protein folding (46) and has been extensively used for studying the biophysical problems related to protein-protein (47) and protein−DNA interactions (42,43,48–53). Further details of the protein model with explicit form of the potential energy functions are given in the supplementary text.

### DNA model

For DNA, we adopted 3SPN.2C coarse-grained model of DNA developed in de Pablo’s group, where each nucleotide is represented by three spherical beads: phosphate, sugar, and a nitrogenous base (54). Each bead is placed at the geometric center of the corresponding moiety. The model successfully captures the correct structural properties of DNA (55). For instance, the model accurately estimates structural features of DNA such as helix width, base-pair rise, number of base-pair per turn and widths of major and minor grooves. The results agree well with the experimental values. Special emphasis was given to reproduce the mechanochemical properties of DNA such as sequence dependent persistence length and flexibility of double-stranded DNA, prediction of melting temperature and estimation of DNA hybridization rate constants for varying sequences under different ionic concentrations. The complete details of the DNA energetics are elaborately described in the supplementary information along with all the reference parameter values listed in Supplementary Tables S1–S7.

To investigate the nonspecific DNA search process by DBPs, we incorporated the following two potential energies to study the nonspecific interactions between protein and DNA: (i) the electrostatic interactions between negatively charged phosphate beads and charged amino acids (Arg, Lys, Glu, Asp), and (ii) the repulsive excluded volume interactions between protein residues and DNA beads. We assigned a unit negative negative charge on both Asp and Glu residues and a unit positive charge is placed on each bead representing Arg and Lys amino acid residues. A negative charge of 0.6 is assigned to each phosphate DNA bead in order to take into account the effect of counterion condensation. The electrostatic interactions were modelled using Debye-Hückel potential that accounts the salt effect. The effective strength of electrostatic interactions between charged beads of protein and DNA is scaled by a factor of 1.67 to bring the local charge of phosphate beads back to −1, as used previously (56,57). It is important to note that the Debye-Hückel theory is valid only for low salt condition and does not hold for an ionic concentration greater than 0.5 M (45). Despite the limitations, the Debye-Hückel potential has been successfully used to investigate protein−DNA recognition (42,48–53,57), modelling specificity in protein−DNA interactions guided by binding assay and structural data (58), rotational coupled sliding of proteins around DNA (59), protein binding induced DNA bending (60) and free energy landscape nucleosome unwrapping (56).

### Designing 336-bp circular DNA conformations for various Δ*Lk* values

We generated 336-bp minicircle DNA with different helical twists using the Nucleic Acid Builder (NAB) module implemented in AMBERTOOLS16 (61). NAB requires the following input parameters to build DNA minicircles: (i) the number of base-pairs (*N*_bp_) and (ii) the degree of DNA supercoiling. The second parameter is defined by the change in linking number Δ*Lk* and is mainly responsible for generating superhelical stress in a circular DNA conformation. A uniform rise of 3.34 Å and a helical turn of 10.5 base-pair were used all throughout. Therefore, for a relaxed 336-bp DNA minicircle, the total number of helical turns will be 336/10.5 = 32. The supercoiling in the minicircle DNA is introduced by the formula *N*_bp_/10.5 + Δ*Lk*. Positive and negative values of Δ*Lk* represent the number of extra helical turns added or subtracted relative to the total number of turns in the relaxed minicircle DNA. Thus, positive values of Δ*Lk* produce over-twisted DNA, whereas negative Δ*Lk* values generate under-twisted DNA. In this study, we build four different 336-bp negatively supercoiled (underwound) DNA minicircle with Δ*Lk* = −4 to −1 (*Lk* = 28–31) and five different 336-bp positively supercoiled (overwound) DNA minicircle with Δ*Lk* = +1 to +5 (*Lk* = 33–37).

### Simulation protocol

We performed two different sets of simulations: (i) simulation of DNA minicircle alone and (ii) simulation of supercoiled DNA in presence of protein.

#### Simulation of DNA minicircle

We started by simulating a 336-bp DNA minicircle conformation with various Δ*Lk* values ranging from −4 to +5. We placed the minicircle DNA at the center of a cubic box of dimension 600 Å^3^ with periodic boundary conditions. The time evolution of the system was studied using Langevin dynamics with friction coefficient γ = 0.01 kg/s and temperature 300 K. It should be noted that at this temperature, the supercoiled DNA does not melt into separate strands in our simulations. This is because the simulation temperature is substantially lower than the predicted melting temperature (*T*_m_ ≈ 381 K) of our 336 bp minicircle sequence at 140 mM salt condition following the phenomenological equation given in (62). Another important fact is that the system is not symmetric around Δ*Lk* = 0, which could be an artifact of the small size of the DNA plasmid as well as the DNA sequence, which may play a significant role in forming the supercoiled structures. At each Δ*Lk* value, we performed 20 independent simulations of 2 × 10^8^ MD steps long at a physiological salt condition of 140 mM. During the simulations, the under-/overwound DNA structures release their torsional strain and adopt different DNA conformations (see Supplementary Video S1 for conversion of minicircle DNA to supercoiled DNA). The conformational heterogeneity of the supercoiled DNA captured by our model is analysed by estimating the population of diverse supercoiled DNA structures and compared with the results obtained from all-atom simulations (44). In order to assess the convergence of our MD simulations, we probed the root-mean-square deviation (RMSD) of the minicircle DNA, the time evolution of which (see Supplementary Figure S1) clearly suggests that the DNA conformation reaches equilibrium within 1.2 × 10^8^ MD steps as shown for Δ*Lk* = +3.

#### Simulation of supercoiled DNA in presence of protein

To investigate the differential protein dynamics on supercoiled DNA, we performed extensive molecular simulations of various supercoiled DNA in presence of protein molecule. The most preferred supercoiled conformations of DNA at various Δ*Lk* were selected (see Supplementary Figure S2) and placed with the protein molecule inside a cubic simulation box of dimension 600 Å^3^. Initially, the protein and DNA molecules are kept at 50 Å away from each other. For every Δ*Lk* values, we performed 30 independent simulations each of 1 × 10^8^ MD steps long at an ionic concentration of 140 mM.

### Algorithm for detecting position of juxtaposition sites

The supercoiled DNA buckles to adopt plectoneme conformations in order to release torsional stress. In such plectoneme conformations often distant DNA sites come spatially close to each other, commonly referred as juxtaposition sites. To detect the position of juxtaposition sites in a supercoiled DNA plectoneme, we used the algorithm provided by Desai *et al.* (63), which relies on the spatial distance between two DNA sites. The algorithm proceeds as follows:

- Find the center of mass of each DNA base-pair (bp).
- Loop over all DNA base-pairs starting from 1 to 336.
- Calculate the distance between the current bp (*i*) and all other bp’s (*j*) along the DNA beyond a cut-off of *N*_*c*_ bp. In other words, find the distance between *i*^*th*^ and *j*^*th*^ bp, for all *j* > *N*_*c*_.
- If any distance (*d*_*i*_) is less than a cut-off distance *d*_cutoff_, i.e., *d*_*i*_ < *d*_cutoff_, then record the first bp index as the beginning position of the juxtaposition site.
- Next, we proceed to detect the end position of the juxtaposition site. After detecting the beginning position of juxtaposition site, we skip the next *N*_*c*_ base-pairs along the DNA and identify the bp index that is closest to the beginning position. This closest bp index is defined as the end position of the juxtaposition site.

This algorithm is able to detect multiple juxtaposition sites along the DNA in supercoiled structure. In our calculations, we choose *N*_*c*_ = 50 bp and *d*_cutoff_ = 60 Å. We also checked that the precise choice of these parameters does not significantly alter the results.

## RESULTS AND DISCUSSIONS

### Conformational diversity of supercoiled DNA

**Figure 1.**
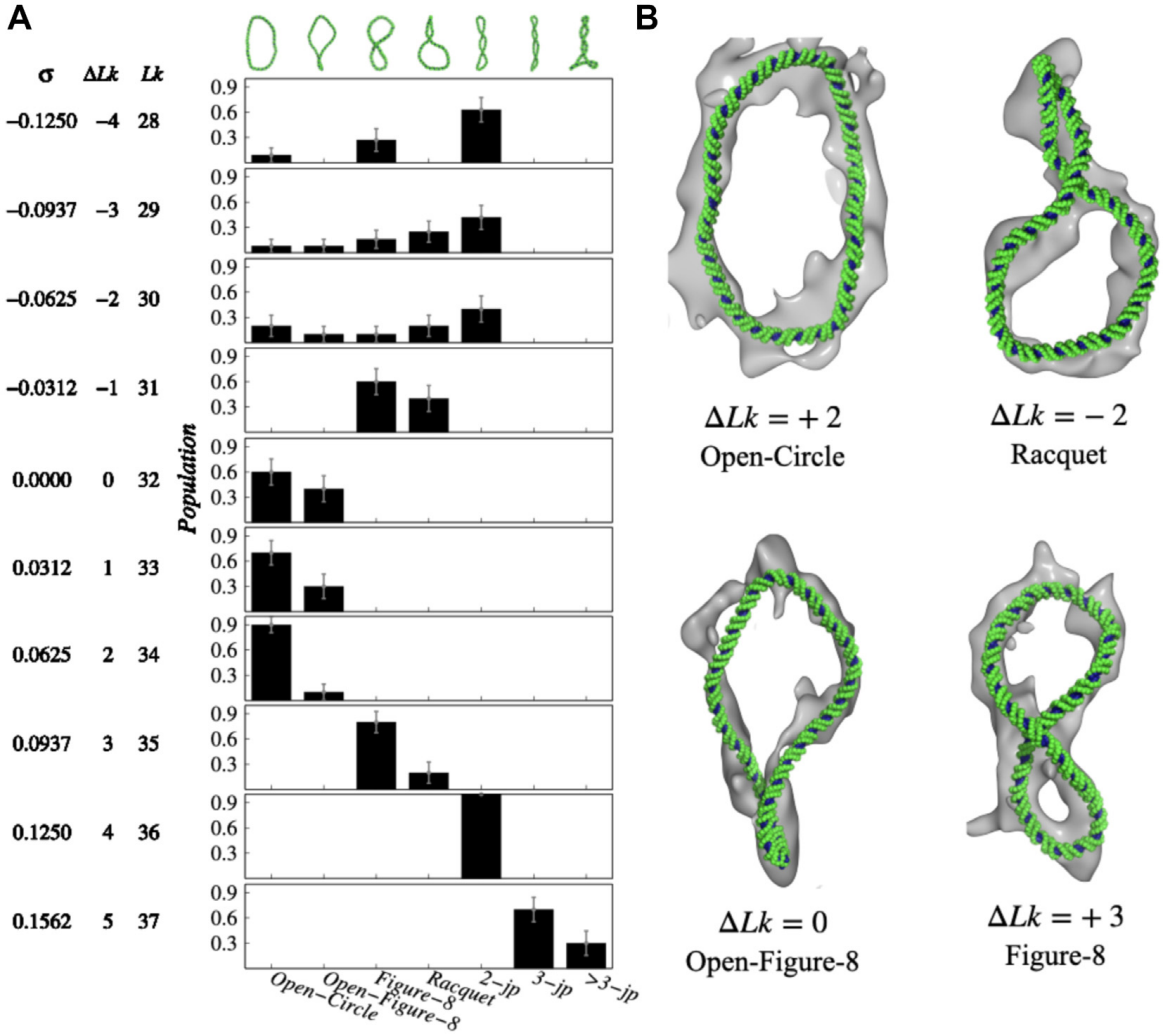
Effect of supercoiling on the 336 bp minicircle DNA structure. (**A**) Population of commonly observed supercoiled DNA structures obtained from the simulation of a 336 base-pair minicircle DNA at different Δ*Lk* values. The commonly observed structures are Open Circle, Open-Figure-8, Figure-8, Racquet and supercoiled structure with 2, 3 and more than 3 juxtaposition (*jp*) sites, each of which are shown at the top of panel A. *Lk*, Δ*Lk* and superhelical density (σ) for each minicircle are shown on the left side (see supplementary text for definition). (**B**) Comparison of cryo-ET structures and the equivalent conformations observed in our coarse-grained MD simulations. Examples from negatively supercoiled (Δ*Lk* = −2), positively supercoiled (Δ*Lk* = +2, +3) and relaxed (Δ*Lk* = 0) DNA structures are shown. Structures generated from coarse-grained MD simulations are shown in double-stranded DNA backbone traces (green color) and the cryo-ET structures are depicted in grey color. The cryo-ET data are obtained from EMDB database under the accession code EMD-6462 (44) and the corresponding structures are generated using the Mol* viewer (95).

Coarse-grained simulations of DNA using 3SPN.2C model describes DNA nucleotides at a near atomistic resolution (64), yet provides the requisite computational speed and efficiency to probe long timescale (ms) structural and functional dynamics of DNA during protein−DNA recognition (42,45,50–53,57). The relatively high resolution and inclusion of important degrees of freedom of the DNA nucleotides are helpful in characterizing specific protein−DNA interactions and capturing alterations in local DNA geometry. These advantages led us to test whether the model affords to explore structural diversity of supercoiled DNA, which can be used further as a basis to probe their interactions with DBPs searching for their specific DNA sites. To begin with, we considered a DNA minicircle of 336 bp. The length presents a representative of supercoiled DNA loops found in nature (44,65–68). The DNA sequence used in this study is same to the one reported in previous cryo-ET experiments (44,69) (see supplementary materials), allowing us to directly compare our results with that of the cryo-ET experiment. In its relaxed form, the 336 bp DNA minicircle shows a helical repeat of ∼10.5 bp/turn, and therefore both the strands wrap around each other 32 times, defining the linking number *Lk* = *Lk*_0_. Any deviation (Δ*Lk* = *Lk* − *Lk*_0_) in *Lk*_0_ results in an overwound or underwound DNA structure under a given torsional stress. We generated four different negatively supercoiled (underwound) minicircle DNA with Δ*Lk* = −4 to −1 (*Lk* = 28–31) and five different positively supercoiled (overwound) minicircle DNA with Δ*Lk* = +1 to +5 (*Lk* = 33–37) by modulating Δ*Lk* values. The corresponding superhelical density (σ = Δ*Lk*/*Lk*_0_) covers a wide range of values ranging from −0.1250 to +0.1562. We simulated all these structures starting from a completely circular form for a long timescale of 2 × 10^8^ MD steps (20 independent runs at each *Lk* value) at a physiological salt concentration of 140 mM. During the simulations, the underwound and overwound DNA topoisomers release their torsional stress and intertwined to adopt different DNA plectoneme structures. Due to intertwining, sequentially distant DNA sites appear close to each other, referred to as juxtaposition sites. In Figure 1A, we present the population of seven distinct supercoiled and plectoneme structures encountered in our simulations, namely open circle, open figure-8, figure-8 like structure, racquet, and structures with 2, 3, and more than 3 juxtaposition sites. The structural diversity observed in our simulations is consistent with a previous atomistic simulation of a similar length of DNA minicircle. Our result suggests that with increasing Δ*Lk* values (high positive and negative values), the structures are more intertwined. In contrast, circular form is prevalent for Δ*Lk* = −2, 0, +1 and +2 values. Ideally, small DNA minicircles should adopt a perfectly round shape in order to minimize the additional energy cost for local DNA bending. However, the ellipticity of small DNA minicircles of base-pairs 94−158 was measured and found to lie between 1.1 and 1.5 (70–72). The deviation from perfectly round shape was attributed to the appearance of hyperflexible kinks within the DNA (72) (see Supplementary Figure S3). To validate our model, we analyzed the shape of open circular forms of DNA observed in our simulations and estimated the ellipticity for 336 base-pair DNA that ranges between 1.26 and 1.56, suggesting a good agreement with the reported values (44). We also find significant overlap between various supercoiled DNA structures obtained in our simulations and predicted from available cryo-ET data (44). The results are presented in Figure 1B that shows the agreement between the two. We also simulated a double-length (672 bps) minicircle DNA to explore the length dependency of supercoiled DNA structures. The structures in Supplementary Figure S4 suggest that double-length minicircle DNA with equivalent supercoiling (Δ*Lk* = −8) to that of 336 bp minicircle DNA (Δ*Lk* = −4) appeared highly writhed. In comparison, the 336 bp minicircle DNA exhibited both open and writhed conformations (Figure 1A). The result is consistent with previous cryo-ET and all-atom study (44). We also explored different supercoiled DNA structures for 672 bp minicircle with higher negative (Δ*Lk* = −7, −9) and positive (Δ*Lk* = +6) supercoiling (Supplementary Figure S4). Taken together, the consistency between our results and previous cryo-ET and atomistic simulation studies (44) in capturing the structural variability of supercoiled DNA validates our DNA model and provides confidence to use the same for exploring the role of supercoiled DNA topology in protein−DNA recognition.

### Nonspecific protein search on linear DNA: study of three architectural proteins

Before proceeding further with investigating the protein−DNA recognition on supercoiled DNA, it is desirable to test the validity of our protein model as well. As mentioned in the Materials and Methods section, we used a minimalistic *C*_α_ model for a protein that can capture the essential conformational features of a protein with a reduced degree of freedom (46). In terms of computational efficiency, such a model is beneficial, particularly in the absence of any noticeable dynamical changes in protein conformation triggered by nonspecific interaction. Therefore, for a folded protein interacting with DNA, it is more important to tune their intermolecular interaction strength in order to precisely capture the mobility of the protein on DNA and recognition of the target DNA site. To test if our model has achieved that, we first studied the diffusion of three architectural proteins on a linear 200 base-pair long DNA segment. Architectural proteins are highly abundant DNA binding proteins in prokaryotes and archaea that preferentially bind with supercoiled DNA and are involved both in regulating the gene expression and shaping the chromatin. This class of protein binds to DNA in a sequence-specific or nonspecific manner and helps in maintaining the genome by introducing bends in the DNA (73). The three architectural proteins that we selected are (i) Fis, a homo-dimer of 98 residue subunits from *E. coli*, that bind through a helix-turn-helix motif into the DNA major groove. (ii) HU, which is a heterodimer of 90 residue subunits from *E. coli*, which contains long beta-ribbon arms that inserts into the DNA minor groove, and (iii) Nhp6A, which is a 93 residue long monomeric protein from Saccharomyces cerevisiae that contains a high mobility group B domain. Nhp6A binds to DNA minor groove, along with an N-terminal flexible tail that binds to the DNA major groove on the opposite side. These three proteins are selected because data corresponding to their one-dimensional diffusion coefficients (*D*_1_) are available from a single-molecule fluorescence imaging study of their binding dynamics with extended DNA (74). Here, we measured *D*_1_ for the same three proteins from their mean square displacements (MSD) on DNA by performing twenty independent 1 × 10^8^ MD steps long simulations for each protein in the presence of a 200 base-pair nonspecific linear DNA stretch at a physiological salt concentration of 140 mM. Our results, presented in Supplementary Figure S5, suggest that diffusion of HU is the fastest followed by Nhp6A and Fis. The result is in agreement with the observed trend in diffusion coefficient measured by Kamagata *et al.* (74), indicating the suitability of the present computational framework for investigating protein mobility on DNA. The study by Kamagata *et al.* (74) further identified that Fis translocation on extended DNA is mechanistically different from that of HU and Nhp6A. Unlike minor groove binder proteins HU and Nhp6A, Fis, which is a major groove binder protein, exhibits two distinct sliding modes during its mobility on DNA (74,75). The values of *D*_1_ for fast and slow modes were estimated as 0.19 ± 0.02 and 0.007 ± 0.006 μm^2^/s (74) on linear DNA, suggesting ∼27 times enhancement of protein diffusivity in the fast sliding mode. The same measured from our simulation suggests ∼34 times enhancement in diffusivity caused by fast sliding mode, indicating an excellent qualitative match with the experimental observations. In slow mode, Fis is stationary and forms a nonspecific complex with DNA causing approximately 55° curvature in the DNA backbone.

### Fis diffuses differently on linear and supercoiled DNA

**Figure 2.**
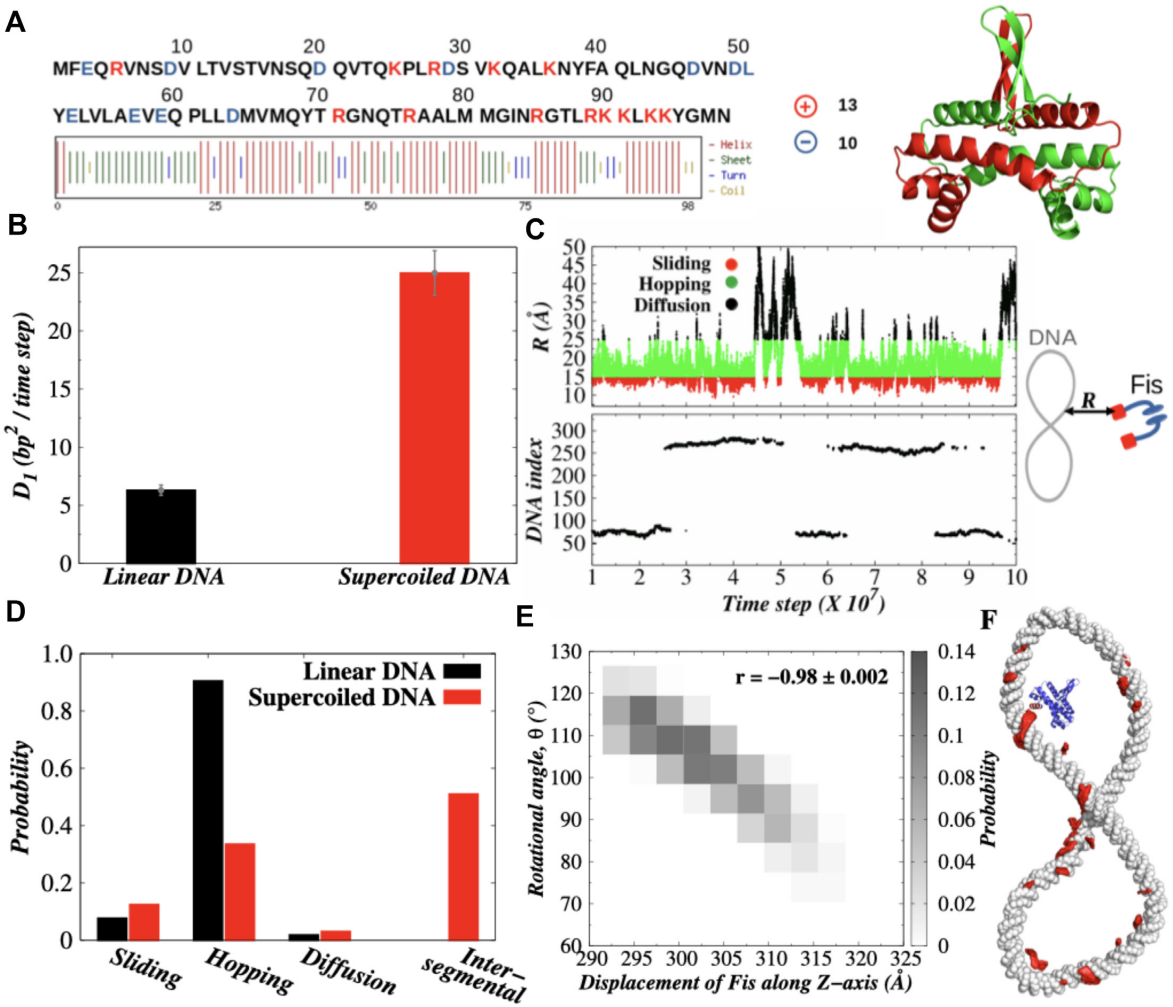
Characterization of protein diffusion on linear and supercoiled DNA. (**A**) Sequence and secondary structure features of Fis protein. Positively (negatively) charged residues are shown in red (blue). *E. coli* Fis homo-dimer with the two chains shown in different colors. (**B**) One dimensional diffusion coefficient (*D*_1_) of Fis protein on linear DNA and supercoiled DNA (Δ*Lk* = −1). (**C**) A representative MD trajectory of Fis diffusion on supercoiled DNA. Top panel: time series of distance from the center-of-mass of any one recognition helix of Fis protein to the center of the closest DNA base-pair (*R*, shown schematically on the right side); Bottom panel: binding position of the recognition helix of Fis protein on DNA. In the top panel the dots are colored according to the criteria defined in the supplementary text for sliding (red), hopping (green) and 3D diffusion (black). (**D**) Propensity of different search modes, namely sliding, hopping, 3D diffusion and intersegmental transfer of Fis protein on linear and supercoiled (Δ*Lk* = −1) DNA. (**E**) Probability histogram of the correlation (denoted by *r*) between transversal (Z-axis) and rotational (θ) motion of Fis protein in supercoiled DNA. (**F**) Sample trace (red color) of rotation-coupled sliding of Fis protein along the major grooves of supercoiled DNA.

We selected Fis (shown in Figure 2A) as a candidate to study protein diffusivity on supercoiled DNA in the rest of the study. Interestingly, we find the mobility (*D*_1_) of Fis increases ∼4 times on supercoiled DNA with Δ*Lk* = −1 compared to that on linear DNA stretch (see Figure 2B). Upon probing the underlying translocation mechanism following the prescription given in supplementary text (and depicted pictorially in Figure 2C) for identifying different modes of nonspecific protein−DNA interactions, we observe significant differences in propensities of various transport modes between linear and supercoiled DNA topologies. The corresponding result is presented in Figure 2D, which suggests that sliding propensity on supercoiled DNA with Δ*Lk* = −1 enhances more than ∼1.6 times compared to that on linear DNA. During sliding, the protein rotates around the DNA major groove, which is coupled with simultaneous advancement along the DNA contour. A high correlation (98%) between the rotational degree and displacement along the DNA contour as shown in Figure 2E confirms the rotation coupled sliding dynamics of Fis on the supercoiled DNA. This is in agreement with previous experimental observation on Fis diffusing on extended DNA (74). The pathway of Fis during rotation coupled sliding is shown in Figure 2F following the footprints of Fis obtained from multiple simulation trajectories.

**Figure 3.**
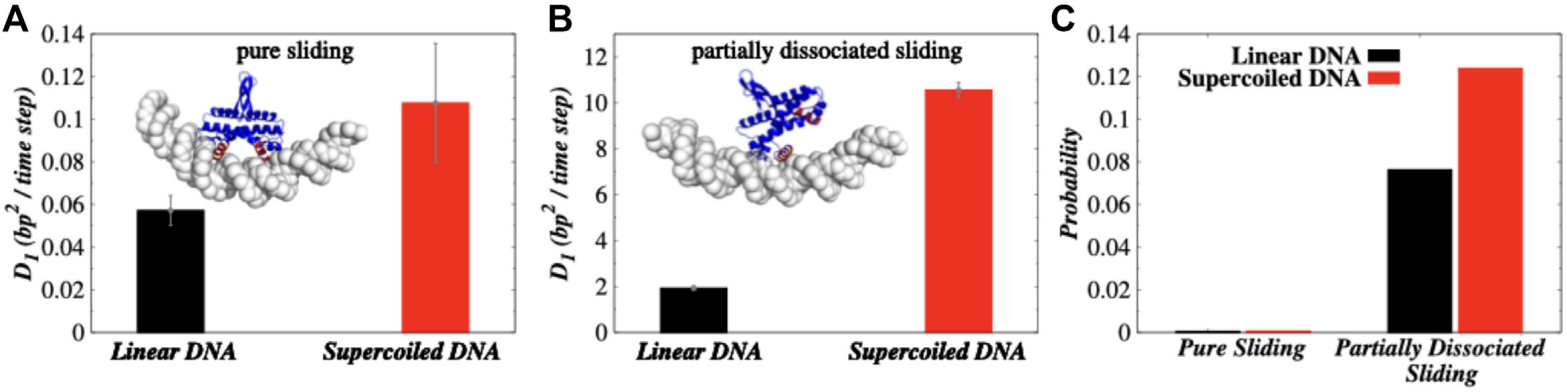
Fis diffusion mechanism on linear and supercoiled DNA. 1D diffusion coefficient (*D*_1_) of Fis protein on linear DNA and supercoiled DNA (Δ*Lk* = −1) under (**A**) pure sliding and (**B**) partially dissociated sliding. The inset for pure sliding shows that both the recognition helices (colored in red) of Fis protein are positioned inside adjacent DNA major grooves, whereas the inset for partially dissociated sliding shows that any one recognition helix of Fis scans the DNA major groove and other remains dissociated from the DNA surface. (**C**) Propensity of pure sliding and partially dissociated sliding on linear DNA and supercoiled DNA (Δ*Lk* = −1).

Further structural analysis of the sliding mode suggests that two different sliding modes are possible in Fis. In one, both the recognition helices of homo-dimeric Fis are positioned inside adjacent DNA major grooves (see Figure 3A, inset), causing a severe compression of the intermediate minor groove. In another mode, the protein remains in a partially dissociated state (Figure 3B, inset), where one of the two recognition helices scans the DNA bases inside a major groove and the other remains dissociated from the DNA surface. We notice that *D*_1_ in the second mode of sliding is ∼100 times faster than the first mode (see Figure 3A, B) on supercoiled DNA. A higher propensity of this fast sliding mode (62%, see Figure 3C) on supercoiled DNA partly contributes towards overall higher *D*_1_ as observed in Figure 2B compared to linear DNA. In comparison to sliding, the hopping mode, which is known as a relatively faster mode of translocation compared to sliding dynamics, exhibits a lower propensity on supercoiled DNA (Figure 2D). This could be disadvantageous for a DBP searching for its DNA binding site on a supercoiled DNA. However, our result suggests supercoiled DNA promotes faster protein diffusion by populating an alternative transport mode in Fis namely the intersegmental transfer (Figure 2D).

Unlike a linear DNA stretch, in supercoiled topology, a change in linking number may induce buckling in the DNA conformation to adopt plectoneme conformations. Because of buckling, sequentially distant DNA segments come close to each forming a juxtaposition site. A diffusing protein at such juxtaposition sites can sense both the DNA segments and may jump from one segment to another, resulting in bypassing scanning a long DNA stretch. The mechanism, known as intersegmental transfer has been previously confirmed by both experimental (76–78) and *in silico* studies (79–81). To check if this is the case for Fis diffusing on supercoiled DNA (Δ*Lk* = −1), we monitored the positions of Fis and DNA juxtaposition site during intersegmental transfers and presented them together in Supplementary Figure S6. We find a strong correlation between the two parameters, indicating Fis is positioned suitably at the DNA juxtaposition site for intersegmental transfer. The protein senses two spatially close DNA segments and forms a transient bridged complex with both the DNA segments through nonspecific electrostatic interactions (see Supplementary Figure S7) before moving completely to one segment. We find Fis scans ∼69% of its total visited DNA bases through intersegmental transfer, highlighting the importance of this transport mode in regulating protein search for its binding site on supercoiled DNA.

### Fis displays faster diffusion on negatively supercoiled DNA compared to positive supercoil topology

**Figure 4.**
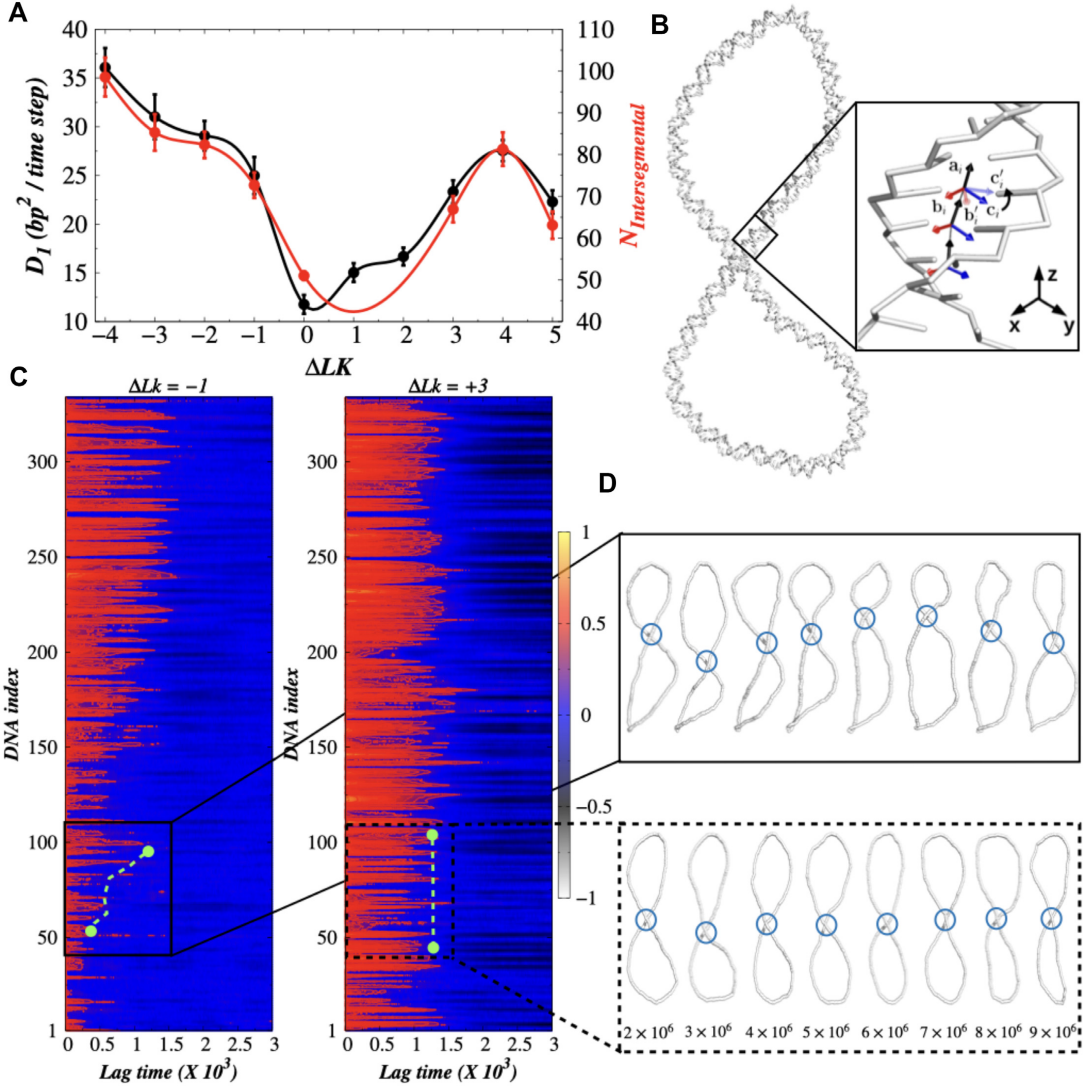
Protein diffusion and twist propagation of supercoiled DNA. (**A**) 1D diffusion coefficient (*D*_1_, black line) and the number of unique intersegmental transfer (*N*_*Intersegmental*_, red line) of Fis protein as a function of change in linking number Δ*Lk*. (**B**) Schematic representation for the calculation of twist propagation in which the vector $\boldsymbol {c_i}$ is obtained from the relation $\boldsymbol {c_i} = \boldsymbol {a_i} \times \boldsymbol {b_i}$, where $\boldsymbol {a_i}$ defines the unit vector connecting the mid-points of two consecutive bases and $\boldsymbol {b_i}$ represents the binormal vector. (**C**) DNA twist propagation along arrays of DNA base-pairs. Autocorrelation of *C*(*t*, *i*) landscape for arrays of DNA base-pairs with Δ*Lk* = −1 and +3. The index of each DNA base in the array is marked on the y-axis. The green dotted line indicates the propagation of twist within DNA index 50−100. (**D**) The snapshots, which were sampled at different MD time steps (highlighted region), represent the displacement of juxtaposition site at Δ*Lk* = −1 and Δ*Lk* = +3 in upper and lower panel respectively. The position of juxtaposition site on each supercoiled DNA snapshot is marked as blue circle.

Having seen that supercoiled DNA topology at Δ*Lk* = −1 promotes intersegmental transfer in Fis and thereby facilitates their mobility on DNA, we moved to investigate if this is a trend general to all degrees of supercoiling and how important the intersegmental transfer mode is in regulating the protein diffusion on supercoiled DNA. For this, we evaluated the one-dimensional diffusivity of Fis on different supercoiled DNA structures and the number of the unique intersegmental transfer performed by Fis on each supercoiled DNA topology. Our result is presented in Figure 4A. The result shows two interesting features: (i) both parameters, namely, the unique number of intersegmental transfer and the one-dimensional diffusivity of Fis vary identically with Δ*Lk*, signifying that intersegmental transfer is the primary determinant of protein diffusivity on the supercoiled architecture of DNA. (ii) Among the plectoneme structures with the same number of juxtaposition sites, we observe a higher diffusivity of the protein on negative plectonemes compared to their positive counterparts. We also examined the one-dimensional diffusivity of other architectural proteins and calculated *D*_1_ for four proteins, namely HU, IHF, Nhp6A and Sso7d (see Supplementary Figure S8) for all degrees of supercoiling. Among these proteins, IHF is a heterodimeric protein from *E. coli*, which is a homologue of HU protein and Sso7d is a monomeric protein from *Sulfolobus solfataricus*, which binds to DNA by placing a triple-stranded β-sheet across the DNA minor groove. We find that the trend of *D*_1_ with respect to the degree of DNA supercoiling is robust irrespective of the molecular features of the interacting protein. Therefore, clearly both of our observations require further discussions in order to elucidate their importance. Our result exhibits rise in protein diffusivity (*D*_1_ in Figure 4A) with higher linking numbers (Δ*Lk* = +1 To +5). This is due to the fact that with increasing Δ*Lk*, buckling of DNA conformations increases resulting in forming multiple juxtaposition sites. The diffusing protein performs a higher number of intersegmental jumps as and when visit the juxtaposition site during their scanning of DNA contour, leading to a rise in overall *D*_1_ of the protein. However, this does not illustrate the increasing trend of *D*_1_ for Δ*Lk* = −2 to −4 as the corresponding DNA plectonemes feature two juxtaposition sites (*Wr* ∼ −2, see Supplementary Figure S9). What causes an increase of ∼24% in *D*_1_ even though the number of juxtaposition sites remains the same with Δ*Lk*? Likewise, it also remains unclear why *D*_1_ of Fis is higher on negative plectonemes compared to positive plectoneme structures having an identical number of juxtaposition sites. For example, *D*_1_ of Fis is $\sim 7\%$ higher on DNA with Δ*Lk* = −1 compared to Δ*Lk* = +3, although both have one juxtaposition site (see Supplementary Video S2 and S3 for the diffusivity of Fis on supercoiled DNA with Δ*Lk* = −1 and +3 respectively). Similarly, both the plectoneme conformations corresponding to Δ*Lk* = −2 and Δ*Lk* = +4 feature two juxtaposition sites each but Fis moves $\sim 6\%$ faster on the former. The difference in *D*_1_ of Fis between Δ*Lk* = +4 and Δ*Lk* = −4 is even higher (∼31%). Such differences in the diffusivity of Fis is also observed for the longer length of supercoiled DNA (672 bp). For example, we find that *D*_1_ in negatively supercoiled DNA of length 672 bp (Δ*Lk* = −7, *D*_1_ = 27.41 ± 1.13 bp^2^/time step) is ∼1.6 times faster compared to the positively supercoiled DNA (Δ*Lk* = +7, *D*_1_ = 17.21 ± 1.05 bp^2^/time step) with the same number of juxtaposition sites. Together these observations indicate that the efficiency of intersegmental jumps to speed up the protein diffusion is not only a function of a number of juxtaposition sites, rather may depend also on the conformational differences of positive and negative supercoiled DNA conformations that arise with the change in linking number.

### Protein dynamics is correlated with torsional behaviour of supercoiled DNA

From the relation Δ*Lk* = Δ*Tw* + Δ*Wr*, it is evident that the impact of change in linking number is reflected by the changes in a twist (Δ*Tw*) and writhe (Δ*Wr*). Supplementary Figure S10 describes twist as a function of linking number that suggests a noticeable change in average twist per DNA base-pair with the change in linking number from −4 to −1. Interestingly, Δ*Wr* in this regime remains approximately invariant (same number of juxtaposition sites) and changes only by one unit for Δ*Lk* = −1. This suggests that the DNA conformations corresponding to Δ*Lk* = −1 to −4 differ from each other primarily in terms of Δ*Tw*. The effect is entirely different for changes in linking numbers from +3 to +5. DNA conformations corresponding to intermediate Δ*Lk* = 0, +1 and +2 adopt relaxed circular shape without any intertwining (no juxtaposition site). The number of intersegmental jumps is minimum on these structures, leading to the slowest protein diffusivity (see *D*_1_ in Figure 4A). For Δ*Lk* = +3 to +5, intertwining increases with increasing Δ*Lk*, suggesting the change is primarily regulated by Δ*Wr* (number of juxtaposition sites increases) while Δ*Tw* does not vary much. Comparing both under and overwound plectoneme structures, it is evident that the differences in protein diffusivity among the plectoneme structures with an identical number of juxtaposition sites (fixed Δ*Wr*) are governed by the change in torsional behaviour (Δ*Tw*) of the supercoiled DNA upon a change in linking number. Therefore, we move to probe the torsional behaviour of different plectoneme structures with a same number of juxtaposition sites. We estimated the torsional stress (DNA twist) propagating along the DNA by computing the autocorrelation of $C(t,i) = \langle \boldsymbol {c}_i(0) . \boldsymbol {c}_i (t) \rangle$ between the normal vector $\boldsymbol {c}_i$ at the beginning (*t* = 0) and after time *t* during the simulation (82). Vector $\boldsymbol {c}_i$ is estimated from $\boldsymbol {c}_i = \boldsymbol {a}_i \times \boldsymbol {b}_i$, where $\boldsymbol {a}_i$ defines the unit vector connecting mid-point of *i*th base-pair to the mid-point of *i* + 1^*th*^ base-pair and $\boldsymbol {b}_i$ represents the binormal vector (see Figure 4B). The angular brackets represent the ensemble average over multiple simulation runs. *C*(*t*, *i*) allows us to follow the passage of DNA twist along the length. *C*(*t*, *i*) ≈ 1 indicates DNA segments that are yet to undergo twisting, while *C*(*t*, *i*) < 1 indicates segments that have already undergone twisting. Figure 4C displays the *C*(*t*, *i*) landscape for arrays of DNA base-pairs with Δ*Lk* = −1 and +3. For small or no time lag, all DNA base-pairs exhibit *C*(*t*, *i*) values close to 1, indicating an unchanged torsional behaviour of the DNA. As the time lag increases, the DNA base-pairs undergo twisting that can be observed from the boundary of the blue region. The increase and decrease of the heights of the boundary at different DNA segments suggest propagation of the twist with time, where the slope of the boundary represents the speed of the twist propagation. We observe a significant difference in twist propagation pattern between positive and negative plectonemes. In negative plectonemes, twist propagates at reasonably high speed within different DNA segments. For example, we see a significant rise in slope with increasing lag time within DNA index 50−100 (shown by the green dotted line). Visualization of DNA conformations corresponding to this time interval shows that a DNA site participating in forming juxtaposition site shifts from DNA index 50 to 90. On contrary, the twists in positive plectonemes are more local and propagate extremely slowly. This is confirmed from the *C*(*t*, *i*) landscape of Δ*Lk* = +3 that shows roughly similar heights of the boundary of the blue region over the arrays of DNA base-pairs, for example, within DNA index 50−100 (shown by green dotted line). Corresponding snapshots of DNA conformations do not show significant changes in DNA index participating in forming juxtaposition sites (see Figure 4D). Similar differences between positive and negative plectoneme structures with two juxtaposition sites in Supplementary Figure S11 supports the generality of our observation. The underlying molecular driving force for the higher twist propagation speed in the negative plectonemes originates from the lower stabilities of intra- and inter-stacking of nucleobases as shown in Supplementary Figure S12. For Δ*Lk* < 0, the nucleobases can favourably accommodate twist and maximize the intra- and inter-stacking energies (31) to stabilize the system further. Thus, an alteration in the twist at a site on negative plectoneme structure triggers faster reorientation of the adjacent nucleobases compared to that in positive plectoneme conformations and thereby results in rapid propagation of twist from one site to another in the former. The differences in twist propagation caused by the differences in torsional behaviour of plectoneme conformations is a measure of conformational dynamics in supercoiled DNA, that can be captured from the fluctuation in the position of nucleobases. It is important to mention here that, in eukaryotic chromatin, similar torsional stress can regulate the unwrapping of DNA from nucleosome, which in turn provides access to the genetic information encoded in the DNA. For instance, a recent study (83) has demonstrated that DNA site accessibility in the nucleosome can be achieved through enhancing or suppressing the asymmetric unwrapping of nucleosomal DNA regulated by positive or negative torsional stress. This suggest that the large impact of torsional stress applies the same to both the supercoiled bacterial DNA as well as on the eukaryotic chromatin for the accessibility of target genes.

### DNA supercoiling and juxtaposition site pinning

**Figure 5.**
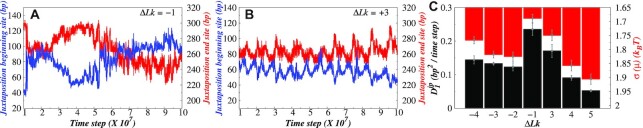
Characterization of juxtaposition dynamics in supercoiled plectoneme. Time-dependent location of the beginning (blue) and end (red) position of the juxtaposition site for (**A**) Δ*Lk* = −1 and (**B**) Δ*Lk* = +3. (**C**) Estimation of juxtaposition diffusivity $D_1^{jp}$ (black bar) and the ruggedness of the potential energy landscape σ(μ) (red bar) of the juxtaposition site as a function of change in linking number Δ*Lk*.

We evaluated the impact of conformational dynamics on negatively and positively supercoiled plectoneme conformations by tracking the position of the juxtaposition site. Figure 5A and B present the change in juxtaposition site position on DNA along the simulation time for both Δ*Lk* = −1 and +3. In general, the impact of twist propagation along the supercoiled DNA can be realized for any DNA site. However, tracking the juxtaposition site position is important as that acts as a launchpad for the protein performing intersegmental jumps. A prominent feature that can be observed in our results is that the position of juxtaposition sites in negative plectoneme is highly dynamic, fluctuating approximately within 80 DNA indices. In comparison, the variation in juxtaposition site in positive plectonemes at Δ*Lk* = +3 reduces roughly to half. To quantify our observation and realize the underlying molecular picture, we further monitored the juxtaposition site positions and estimated the ruggedness of the effective potential around the juxtaposition site, shaped by the torsional behaviour of arrays of DNA base-pairs using the prescription of Putzel *et al.* (84). We partitioned the space into cubic cells of size 10 Å, which is greater than the radius of any nucleobases. The changing position of the juxtaposition site can be mapped into different cells against an effective potential equal to the excess contribution to the diffusing site’s chemical potential ($\mu _i^{cell}$),

(1) $$\begin{equation*}\mu _i^{{\rm cell}} = -k_{\rm B}T . {\rm ln} \bigg (V_{{\rm cell}}^{-1} \int _{{\rm cell} i} {\rm e}^{-\beta U(\vec{r})} {\rm d}\vec{r}\bigg )\end{equation*}$$

where $U(\vec{r})$ is the total potential energy acting on the juxtaposition site at position $\vec{r}$. By discretizing the above integral (see Supplementary text for details), we can calculate the effective potential of each cell and subsequently measure the roughness of the landscape by measuring the corresponding standard deviation σ(μ^cell^). The roughness is plotted against change in linking number in Figure 5C along with the diffusivity ($D_1^{jp}$) of the juxtaposition sites in various plectoneme conformations. Our results capture two important insights: (i) the roughness (σ) of effective potential energy landscape around DNA juxtaposition site shares an inverse relationship with the diffusivity $D_1^{jp}$ of the juxtaposition site, indicating the former as a determinant of the juxtaposition site dynamics. Higher the σ, slower is the diffusion of the juxtaposition site. For DNA plectoneme structures corresponding to Δ*Lk* = −2 to −4, we note that σ decreases gradually and $D_1^{jp}$ increases accordingly, suggesting the conformational dynamics of the supercoiled DNA depends on the torsional behaviour of the DNA bases. The more underwound the conformation is, the higher the speed of twist propagation (and therefore, higher the diffusivity of the position of juxtaposition site) along with the DNA upon alteration of local DNA twist. (ii) Comparing the $D_1^{jp}$ of positively and negatively supercoiled plectoneme conformations with an identical number of juxtaposition sites (for example, comparison between Δ*Lk* = −1 and +3 or −2 to −4 with +4), we find higher ruggedness (σ) of the potential surface around juxtaposition site position in positively supercoiled plectonemes and accordingly reduction in diffusivity of the position of juxtaposition site. σ also increases with an increase in the number of juxtaposition sites (as in Δ*Lk* = +3 to +5), suggesting the juxtaposition sites act as a boundary to the twist propagation in DNA. The higher the number of juxtaposition sites, the smaller is the fragment of DNA segment within which twist can propagate freely, accordingly more pinned is the position of the juxtaposition site. This explains why diffusion of protein is less (see Figure 4A) on high positive plectoneme conformations despite having more juxtaposition sites. On contrary, higher juxtaposition dynamics in negative plectonemes have a clear advantage in transporting the interacting DBPs to newer DNA sites thereby enhancing the effective scanning span of the protein. For example, our simulation with Fis protein suggests that it covers more number of DNA sites on negatively supercoiled plectoneme conformations compared to positively supercoiled plectoneme structures with one juxtaposition site (see Supplementary Figure S13). Thus, the protein diffusivity on supercoiled DNA is largely controlled by the torsional behaviour of supercoiled DNA, which is a prerequisite to rapid and efficient recognition of the target DNA site involved in crucial DNA metabolic processes.

### Simulation results of Fis diffusion on supercoiled DNA agree with a statistical mechanical model

**Figure 6.**
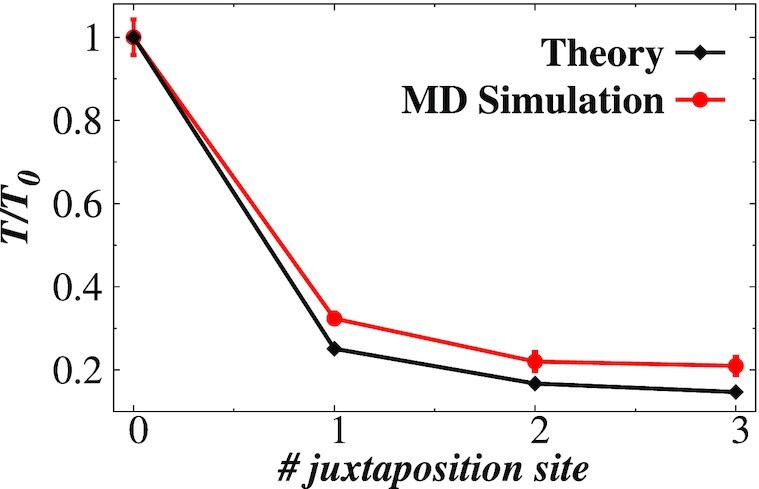
Comparison between theoretical and simulation results. Ratio of the average target search time of Fis protein on supercoiled DNA with (*T*) and without (*T*_0_) juxtaposition site as a function of number of juxtaposition sites obtained from analytical calculations (Equation 2) and MD simulations. The target site is positioned at the site *L* and the juxtaposition sites are placed at *L*/2, (*L*/2, *L*/3), and (*L*/4, 3*L*/8, *L*/2) for supercoiled DNA with one, two, and three juxtaposition sites, respectively. The parameters used for the theoretical calculations are *L* = 336 bp, *k*_off_ = 9 × 10^−5^*s*^−1^, *u* = 10^3^*s*^−1^, *k*_on_ = 112 500 s^−1^ and *k*_*t*_ = 10^6^ s^−1^. The dissociation rate *k*_off_ of Fis protein is adopted from single-molecule experiment (85) and the association rate *k*_on_ is obtained from the experimentally measured dissociation constant $K_{\rm D} = \frac{k_{{\rm off}}}{k_{{\rm on}}} = 0.8$*nM* (86).

To verify the results obtained from our MD simulations, at least in part, we compared the simulation results with an analytical result derived from our previously developed statistical mechanical model of the target search process of proteins on supercoiled DNA (43). To this end, it is noteworthy that the molecular picture revealed by our detailed analysis of the MD simulation trajectories is not straightforward to capture experimentally in active DNA due to the associated difficulties with measuring DNA supercoiling. It is, however, possible to probe the kinetic differences in the target search process of proteins on different plectoneme structures due to the presence of juxtaposition sites and their diffusivity. Our theoretical framework is based on a discrete-state stochastic process that accounts for relevant biophysical and biochemical transitions such as binding, unbinding, and diffusion along with the DNA and analyzes the first-passage events in the system. The model explicitly takes into account supercoiled DNA topologies with an increasing number of juxtaposition sites and evaluates their impacts by calculating the mean first-passage time (MFPT) of reaching the target sites by proteins. Here, we briefly describe the theoretical model and the detailed description of the same can be found in the supplementary text. We consider a single protein searching for its target site on a supercoiled DNA of *L* (=336 bp) binding sites, out of which *L* − 1 sites are considered as nonspecific binding sites and one site is chosen as the target site (placed at the *L*^*th*^ site) at which the protein can bind specifically (see Supplementary Figure S14). The protein starts the search process from the solution phase and can associate to any nonspecific DNA site with the rate *k*_*on*_. In the reverse reaction, the protein can dissociate from any nonspecific DNA site with a rate *k*_off_. Once the protein lands on any DNA site, it can slide along the DNA contour either in forward and backward directions with the diffusion rate *u*. Since the supercoiled DNA topology promotes the transfer of a protein across two sequentially distant but spatially close DNA segments through intersegmental jumps, we, therefore, consider the corresponding transition rate as *k*_*t*_ for intersegmental transfer between juxtaposition sites. In the present study, we adopted the experimentally determined (85,86) association and dissociation rates of Fis protein and calculated the MFPT of reaching the target site on supercoiled DNA with 0, 1, 2 and 3 number of juxtaposition sites. The analytical expression of average target search time is given by

(2) $$\begin{equation*}T = \frac{k_{{\rm off}} L + k_{{\rm on}} [L - S_i(0)]}{k_{{\rm on}} k_{{\rm off}} S_i(0)}\end{equation*}$$

where the auxiliary function *S*_*i*_(0) has a different expression for supercoiled DNA with 0, 1, 2 and 3 number of juxtaposition sites, whose explicit forms are given in the supplementary text. In Figure 6, we present the ratio of the target search times on supercoiled DNA with (*T*) and without (*T*_0_) juxtaposition sites as a function of the number of juxtaposition sites. We find that the results obtained from analytical calculations are in excellent agreement with the results obtained from MD simulations. The result shows that the average target search time decreases exponentially with an increasing number of juxtaposition sites. Noticeably, the analytical model applies for fixed juxtaposition site positions. Therefore, to validate the role of juxtaposition site dynamics, which is found to be modulated by the torsional behaviour of arrays of DNA base-pairs in negatively supercoiled DNA conformations, we performed kinetic Monte Carlo (MC) simulations based on this analytical model. The details are presented in the Supplementary text. Our result in Supplementary Figure S15 shows that the target search time decreases steadily with the increase in the juxtaposition site dynamics as revealed from our MD simulations as well.

### Variation in protein diffusivity on different supercoiled DNA modulates their shape

**Figure 7.**
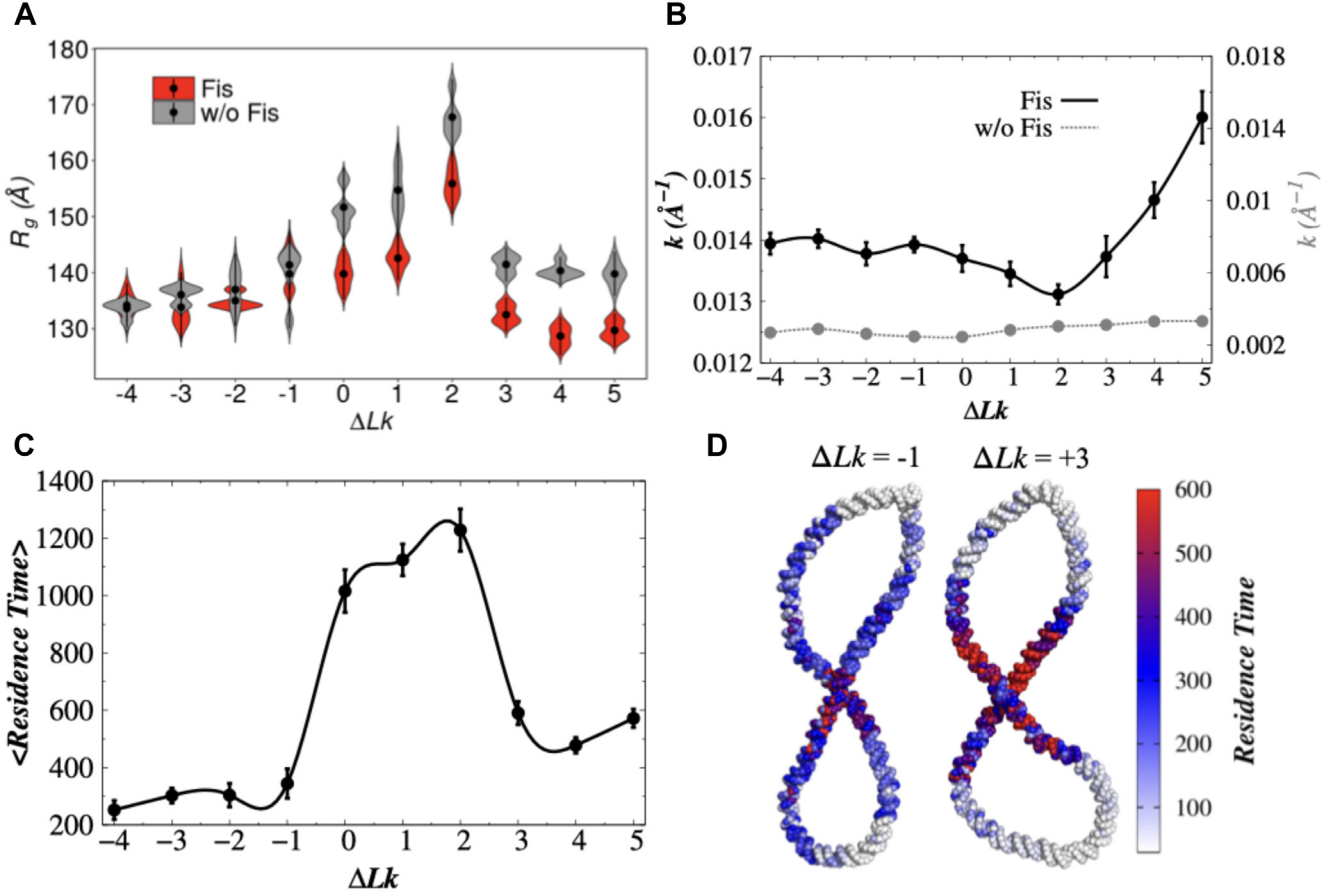
Effect of protein diffusion on the compaction of supercoiled DNA. (**A**) The radius of gyration (*R*_*g*_) of supercoiled DNA in the presence (red) and absence (grey) of Fis protein are shown as a violin plot for each Δ*Lk* value. (**B**) Average curvature (*k*) of DNA at the Fis binding site (black line) are shown with respect to Δ*Lk* values. During these calculations, we identified the Fis binding sites and used them to evaluate the DNA curvature in the absence of Fis protein (grey dotted line). (**C**) Average residence time of Fis protein on supercoiled DNA with different Δ*Lk* values. The residence time is obtained by counting the number of times the protein probed a DNA site during the simulations. (**D**) Schematic representation of the residence time of Fis protein on DNA sites for Δ*Lk* = −1 and Δ*Lk* = +3.

Besides recognition of the target DNA sites, a key aspect in DNA supercoiling is its cellular organization and here we question if the protein diffusivity regulated by torsional behaviour of arrays of DNA base-pairs can influence the overall organization of the genomic material. To investigate the same, we estimated the compactness of DNA conformation through the radius of gyration (*R*_g_) in the presence and absence of the interacting DBP as a function of change in linking number and present the result in Figure 7A. The result suggests that with increasing linking number the compaction in DNA conformations increases (low *R*_g_ values) due to buckling of the structure. DNA with high linking numbers intertwined to form plectonemes that are much more compact compared to the relaxed circular form of DNA. It is interesting to see that the compaction of DNA conformations is higher in the presence of Fis among relaxed and positively supercoiled DNA conformations as evident from lowering of their respective *R*_g_ values compared to when the protein was not present. However, no substantial change in DNA compaction is observed for negative plectonemes in the presence of Fis. To test the robustness of our observation, we further investigated the compaction of DNA conformation in the presence of four other architectural proteins: HU, IHF, Nhp6A and Sso7d (see Supplementary Figure S16) as a function of Δ*Lk*. We find a similar trend in *R*_g_ for heterodimeric proteins HU and IHF to that of Fis. A slightly less pronounced but visible compaction of positively supercoiled DNA conformations are noted for monomeric proteins Nhp6A and Sso7d that have lower propensities for intersegmental jumps compared to the multimeric proteins. The result is consistent with previous observation that nonspecific binding of Sso7d protein induces compaction in only positively supercoiled and relaxed DNA topologies (87) and play a key role in organizing chromatin structure. Our simulation result provides a wealth of additional information regarding the molecular insights into selective compaction of positively supercoiled DNA by analyzing the nonspecific protein−DNA complexes in great detail. Fis for example, in its specifically bound complex (PDB ID: 3IV5) is shown to protrude its two DNA binding helices into adjacent DNA major grooves and bind while imparting a local kink of ∼65° (88). The curved DNA surface brings the DNA bases close enough to the protein binding sites to establish strong enough specific contacts. Likewise, for a stable nonspecifically bound complex with DNA, Fis may impart similar bending in local DNA geometry. To test if this is the case, we measured the average curvature of DNA surface at the nonspecific protein-bound sites and compared that with the same in the absence of protein (from simulations of supercoiled DNA only). The result presented in Figure 7B suggests the DNA curvature in the absence of protein does not vary much with Δ*Lk*. The impact of protein is also insignificant for negatively supercoiled DNA although the curvature increases from Δ*Lk* = 0 to higher positive values. We then measured the average residence time of Fis while performing sliding or hopping on the DNA by monitoring its position to the closest DNA base during our simulations. The results are shown in Figure 7C and schematically in Figure 7D that clearly suggest that the residence time of Fis on DNA is relatively higher while diffusing one-dimensionally on relaxed and supercoiled DNA. The low residence time of Fis on negative plectonemes during its sliding and hopping dynamics is possibly due to the torsional behaviour of nucleobases that triggers faster juxtaposition site dynamics and thereby rapid transport of the interacting DBP along with. In comparison, Fis moves slowly on relaxed and positive plectonemes (consistent with slower *D*_1_ of Fis), providing opportunities to impart local DNA bending to maximize the strength of nonspecific protein−DNA contacts. Such local bending is evident form the snapshots of DNA with and without Fis, as shown in the Supplementary Figure S17 for positive supercoiling (Δ*Lk* = +3, +4). Higher the residence time, higher is the life of such DNA kinks that can effectively reduce the *R*_g_ of overall DNA conformation, explaining why Fis dynamics on DNA may also influence the packaging of genetic material within the nuclear volume.

## CONCLUSION

It has been well accepted that transcription and DNA supercoiling are strongly coupled in a dynamic fashion. Researchers were able to map supercoiling domains in the human genome at a reasonably high resolution to find hundreds of domains with supercoiled DNA topology (89). Most of them are underwound and are actively transcribed regions of ‘open’ chromatin fibers. On contrary, an overwound domain corresponds to transcriptionally inactive chromatin and assumes more compact conformations. The underlying molecular mechanism of the supercoil topology regulated gene expression, however, remains elusive. In this study, we address the issue by investigating how does the supercoil DNA topology regulates the recognition of a target DNA site by a DNA binding protein? We provide a high-resolution computational framework that is carefully tailored to capture the conformational heterogeneity of supercoiled DNA upon a change in linking number and tuned to model protein−DNA interaction effectively. We test our model by comparing the predicted conformational heterogeneity of supercoiled DNA from our model with that of a cryo-ET experiment and a previous atomistic simulation. A good agreement between the two supports suitability in employing the proposed computational framework for probing target search recognition of protein on supercoiled DNA. By performing extensive molecular simulations, we discover that the DNA supercoil topology and protein search process for the target DNA site is closely related. The target search efficiency of the protein was measured in terms of its diffusivity on the supercoiled DNA and its average time taken to reach the target DNA site. A supercoiled DNA often buckles to release excess torsional stress thereby forms different plectoneme structures. Because of buckling, two sequentially distant DNA sites may spatially come close and form a juxtaposition site. It has been suggested previously that proteins having a secondary site to bind with DNA may perform intersegmental jumps at juxtaposition sites that allow the protein to move from one DNA segment to another without scanning the intermediate DNA bases (90). Kinetically intersegmental transport mode is advantageous to globally scan the DNA bases faster. We note that with a higher degree of buckling, the plectonemes feature multiple juxtaposition sites that effectively enhance protein diffusivity on supercoiled DNA. The higher the degree of supercoiling, the faster is the diffusivity of the protein. We validate the result by comparing them with an independent statistical model developed previously by us. The analytical model based on a discrete-state stochastic process takes experimentally reported kinetic rate constants as input and predicts the mean first-passage time (MFPT) of reaching the target DNA sites by the protein in presence of juxtaposition site. An excellent match between the analytical and MD simulation results establishes the accuracy of the latter in capturing protein diffusion on supercoiled DNA.

Our second major observation is that protein diffuses significantly faster on negatively supercoiled DNA compared to positively supercoiled topology having an identical number of juxtaposition sites. The underlying molecular picture suggests that in negatively supercoiled DNA, the underwound topology allows accommodating additional twists at a DNA site. Our study shows that such twist propagates along with the DNA much faster on negatively supercoiled topology compared to its positive counterpart, resulting in faster conformational dynamics in negatively supercoiled DNA. By tracking the juxtaposition site position along the DNA contour we confirmed its dynamic character on negatively supercoiled DNA. In comparison, the position of the juxtaposition site is approximately pinned on positively supercoiled DNA featuring the same number of juxtaposition sites (same writhe). A dynamic juxtaposition site promotes intersegmental jumps of the searching protein at newer DNA sites, allowing a negative supercoil topology to promote intra-domain protein-DNA communications (faster diffusivity). The potential impact may be seen in the expression of the genes, whose activation requires interactions between promoters with distally located enhancers (91,92) mediated by specific transcription factors at the both enhancer and promoter regions. Analysis of Pax6 gene loci indeed suggests that the corresponding segment of chromatin organises such that the active state of the gene features a substantially higher proportion of enhancer-promoter contacts compared to its silenced state (93). We argue that an underwound supercoil topology favours quick communications among such regulatory DNA segments by rapidly diffusing transcription factors due to its distinct torsional behaviour compared to an overwound DNA topology.

The third significant insight revealed from our study is that variation in protein diffusivity on different supercoiled DNA also influences the shape of the latter. We showed that while the presence of protein on negatively supercoiled topology has a negligible impact on its conformation, the presence of protein on relaxed and positively supercoiled DNA topologies induces compaction. The result is consistent with a previous experiment. Our study, however, captures the underlying mechanism that suggests the protein in one-dimensional search mode attempts to bend DNA locally in order to maximize the strength of nonspecific protein−DNA contacts. Since protein diffusion is slower on relaxed and positively supercoiled DNA compared to negatively supercoiled DNA, lifetime of such local DNA bending is longer on the former, causing a compaction of the overall DNA structure. The result explains the role of protein dynamics in differential packaging of genetic material within nuclear volume based on the supercoil topology of the DNA.

In summary, we presented a computational framework that unravels the molecular origin of differences in target search efficiencies of proteins on a different degree of supercoiled DNA topology and plectonemic conformations. The consistency of our results with some of the experimental studies indicates that the proposed mechanism underscores the kinetic advantages of the highly transcribed genes that they enjoy being located preferentially on the chromatin domains with underwound (negatively supercoiled) DNA topology. The clear kinetic advantage of a diffusing protein on negatively supercoiled topology due to the torsional behaviour of the latter may also play a significant role in maintaining genome integrity. A recent study has claimed that mismatch in DNA base-pairs increases the probability of plectoneme pinning (63). The plectoneme pinning propensity is also correlated with the sequence of supercoiled DNA (94). It would be interesting to see how the torsional behaviour of such DNA plectonemes may facilitate recognition of the lesion and employment of the DNA repair proteins. Furthermore, the recent advancement in genome sequencing and microarray analysis have portrayed a complex and dynamic picture of the interplay between *in vivo* gene transcription and DNA supercoiling. Single-molecule studies have the potential to complement these approaches and provide invaluable insights regarding the torsional behaviour of DNA. Our computational framework paves a way to meet the requirement and unravels the molecular picture in detail.

## DATA AVAILABILITY

Simulation data will be made available upon reasonable request.

## Supplementary Material

gkac522_Supplemental_Files
