# Supplementary material for: Torsional behaviour of supercoiled DNA regulates recognition of architectural protein Fis on minicircle DNA
Source: Nucleic Acids Res. 2022 Jun 24;50(12):6671–86. doi: 10.1093/nar/gkac522 (PMC9327302; doi:10.1093/nar/gkac522)
Supplement: gkac522_Supplemental_Files [file gkac522_supplemental_files.zip › Supplementary_Information.pdf]

Supplementary Data

Torsional behaviour of supercoiled DNA regulates recognition of architectural protein Fis on minicircle DNA

Anupam Mondal, Sangeeta and Arnab Bhattacherjee*

School of Computational and Integrative Sciences, Jawaharlal Nehru University,
New Delhi – 110067, India

*E-mail: arnab@jnu.ac.in

This PDF file includes:

Supplementary Text
Supplementary Tables S1 to S7
Supplementary Figures S1 to S17
Supplementary Video Legends
Supplementary References

Protein model

The structure of a protein molecule is designated by a coarse-grained C_α model, where each amino acid residue is represented by a single bead centered on its $\alpha-$carbon (C_α) position. The potential energy function to model the protein energetics includes both the bonded and non-bonded interactions, given by

$$E_{pot}^{protein} = E_{bonded}^{protein} + E_{non-bonded}^{protein} \tag{S1}$$

The bonded potential, $E_{bonded}^{protein}$ is a combination of bond, angle and torsional energies:

$$\begin{aligned} E_{bonded}^{protein} &= E_{bond}^{protein} + E_{bend}^{protein} + E_{torsion}^{protein} \\ &= \sum_{bonds}^{N-1} K_b(r_{ij} - r_{ij}^0)^2 \\ &+ \sum_{angles}^{N-2} K_\theta(\theta_{ijk} - \theta_{ijk}^0)^2 \\ &+ \sum_{torsions}^{N-3} K_{\phi_1}[1 - cos3(\phi_{ijkl} - \phi_{ijkl}^0)] + K_{\phi_2}[1 - cos(\phi_{ijkl} - \phi_{ijkl}^0)] \end{aligned} \tag{S2}$$

where $K_b = 100.0$ kJ/mol/Å^2, $K_\theta = 20.0$ kJ/mol/rad^2, $K_{\phi_1} = 0.5$ kJ/mol and $K_{\phi_2} = 1.0$ kJ/mol. In the equation, the term r_{ij} stands for the instantaneous distance between consecutive bonded beads $i - j$, θ_{ijk} denotes the instantaneous angle between successive bonded beads $i - j - k$ and ϕ_{ijkl} represents the instantaneous torsion angle between consecutive bonded backbone beads $i - j - k - l$. The parameters r_{ij}^0, θ_{ijk}^0, and ϕ_{ijkl}^0 represents the corresponding variables at the native structure.

The non-bonded potential energy function is given by

$$E_{non\text{-}bonded}^{protein} = E_{native}^{protein} + E_{ev}^{protein} + E_{elec}^{protein} \tag{S3}$$

The first term $E_{native}^{protein}$ is the conformational energy that favors the formation of native contacts found in the experimentally determined crystal structure or folded native structure. Here an $i - j$ pair that do not participate in any bonded interactions, is considered to form a "native-contact" if the distance between any two non-hydrogenous atoms of the ith and jth amino acids is within 6.5 Å in the native structure. These native contacts, thus help to maintain the protein native fold during the simulations. Such structure based potential (as originally proposed by Clementi *et al.* (1)) is modelled by a Lennard−Jones (L−J) potential given by,

$$E_{native}^{protein} = \sum_{|i-j|>3}^{native-contacts} \epsilon_{contacts}\left[5\left(\frac{\sigma_{ij}}{r_{ij}}\right)^{12} - 6\left(\frac{\sigma_{ij}}{r_{ij}}\right)^{10}\right] \tag{S4}$$

where $\epsilon_{contacts} = 1.0$ kJ/mol. σ_{ij} is the $C_\alpha - C_\alpha$ distance between $i - j$ pairs that are in contact with each other in the crystal structure and r_{ij} is the same distance in the intermediate structures generated during the simulations.

The second term $E_{ev}^{protein}$ represents the short-ranged repulsive excluded volume interactions between all the non-native C_α pairs and is given by,

$$E_{ev}^{protein} = \sum_{|i-j|>3}^{\substack{non-native \\ contacts}} \epsilon_{repulsions}\left(\frac{\sigma_{ij}}{r_{ij}}\right)^{12} \tag{S5}$$

where $\epsilon_{repulsions} = 1.0$ kJ/mol, σ_{ij} is the sum of the radii between sites i and j that are not forming a native contact and r_{ij} is the distance between them. The repulsion radius of the C_α bead is set to 2.0 Å .

The last term $E^{protein}_{elec}$ is the long-ranged electrostatic interactions between charged amino acid residues and are modelled by Debye-Hückel potential. Arg and Lys residues are assigned a unit positive charge, whereas Asp and Glu residues are assigned a unit negative charge in the model. The Debye-Hückel interaction energy is given by,

$$E^{protein}_{elec} = \sum_{i<j}^{n_{elec}} \frac{q_i q_j e^{-r_{ij}/\lambda_D}}{4\pi\epsilon_0\epsilon(T,C) r_{ij}} \tag{S6}$$

in which the Debye screening length, λ_D is defined as

$$\lambda_D = \sqrt{\frac{\epsilon_0\epsilon(T,C)}{2\beta N_A e_c^2 I}} \tag{S7}$$

Here, q_i and q_j denotes charges on i^{th} and j^{th} charged amino acid beads, r_{ij} is the separation distance between them and n_{elec} is the total number of electrostatic interaction pairs. ϵ_0 and ϵ are the dielectric permittivity of the vacuum and solution respectively. I and e_c denotes the ionic strength of the solution and the elementary charge. N_A is the Avogadro's number and $\beta = \frac{1}{k_B T}$ denotes inverse thermal energy. The dielectric permittivity of the solution $\epsilon(T,C)$ is a function of temperature T and molarity of NaCl, which can be approximated as

$$\epsilon(T,C) = \epsilon(T)a(C) \tag{S8}$$

where

$$\epsilon(T) = 249.4 - 0.788T/K + 7.20 \times 10^{-4}(T/K)^2 \tag{S9}$$

$$a(C) = 1.00 - 2.551C/M + 5.151 \times 10^{-2}(C/M)^2 - 6.889 \times 10^{-3}(C/M)^3 \tag{S10}$$

DNA model

In this study, we adopted 3SPN.2C coarse-grained model of DNA developed in de Pablo's group, where each nucleotide is represented by three spherical beads: phosphate, sugar, and a nitrogenous base (2). Each bead is placed at the geometric center of the corresponding moiety. The potential energy function to model the DNA energetics includes bonded and non-bonded contributions, given by

$$E^{DNA}_{pot} = E^{DNA}_{bonded} + E^{DNA}_{non-bonded} \tag{S11}$$

Bonded potentials

The bonded potential, E^{DNA}_{bonded} is a combination of bond, angle and torsional energies:

$$E^{DNA}_{bonded} = E^{DNA}_{bond} + E^{DNA}_{bend} + E^{DNA}_{torsion} \tag{S12}$$

The bond potential combines both harmonic and anharmonic terms, given by

$$E^{DNA}_{bond} = \sum_i k_b\big(r_i - r_i^0\big)^2 + 100k_b\big(r_i - r_i^0\big)^4 \tag{S13}$$

where $k_b = 0.6$ kJ/mol/Å^2 is the force constant, r_i^0 and r_i are the equilibrium and instantaneous bond lengths for the ith bond.

The angle potential is harmonic, given by

$$E_{bend}^{DNA} = \sum_i k_\theta \big(\theta_i - \theta_i^0\big)^2 \tag{S14}$$

where k_θ represent the force constant for bending which is dependent on the sequence of DNA. The sequence dependent force constant for bending energy at each base-step is shown in supplementary Table S1 that introduce the sequence-dependent flexibility of DNA. θ_i^0 and θ_i are the equilibrium and instantaneous bend angles for bend i.

Supplementary Table S1: Force constants (k_θ) for bending angle potential in 3SPN.2C DNA model.

Angle	Base-Step	k_θ kJ/mol/rad^2	Angle	Base-Step	k_θ kJ/mol/rad^2	Angle	Base-Step	k_θ kJ/mol/rad^2
A–S–P	AA	460	C–S–P	CA	206	S–P–S	AA	355
A–S–P	AT	370	C–S–P	CT	358	S–P–S	AT	147
A–S–P	AC	442	C–S–P	CC	278	S–P–S	AC	464
A–S–P	AG	358	C–S–P	CG	278	S–P–S	AG	368
P–S–A	AA	460	P–S–C	AC	442	S–P–S	TA	230
P–S–A	TA	120	P–S–C	TC	383	S–P–S	TT	355
P–S–A	CA	206	P–S–C	CC	278	S–P–S	TC	442
P–S–A	GA	383	P–S–C	GC	336	S–P–S	TG	273
T–S–P	TA	120	G–S–P	GA	383	S–P–S	CA	273
T–S–P	TT	460	G–S–P	GT	442	S–P–S	CT	368
T–S–P	TC	383	G–S–P	GC	336	S–P–S	CC	165
T–S–P	TG	206	G–S–P	GG	278	S–P–S	CG	478
P–S–T	AT	370	P–S–G	AG	358	S–P–S	GA	442
P–S–T	TT	460	P–S–G	TG	206	S–P–S	GT	464
P–S–T	CT	358	P–S–G	CG	278	S–P–S	GC	228
P–S–T	GT	442	P–S–G	GG	278	S–P–S	GG	165
P–S–P	All	300						

The torsional potential is represented by a Gaussian well, given by

$$E_{torsion}^{DNA} = \sum_i -k_\phi exp\left(\frac{-\big(\phi_i - \phi_i^0\big)^2}{2\sigma_{\phi,i}^2}\right) \tag{S15}$$

where $k_\phi = 7.0$ kJ/mol/rad^2 is the Gaussian well-depth and $\sigma_{\phi,i}$, ϕ_i^0 and ϕ_i are the Gaussian well-width, equilibrium and instantaneous angle for the ith dihedral respectively. The torsional potential applies between torsion angles

formed by phosphate and sugar beads (i.e., P-S-P-S and S-P-S-P).

There is also an added torsional potential function to the DNA backbone, whose functional form is given by

$$E_{\phi,periodic} = k_{\phi,periodic}\big[1 + cos\big(\phi_i - \phi_i^0\big)\big] \tag{S16}$$

where $k_{\phi,periodic} = 2.0$ kJ/mol/rad^2 is the force constant.

All the equilibrium values of bond lengths (r_i^0), bend angles (θ_i^0) and torsion angles (ϕ_i^0) are listed in supplementary Table S2.

Supplementary Table S2: Table of equilibrium bond lengths (r_i^0), bend angles (θ_i^0) and torsion angles (ϕ_i^0) in the DNA model (3). The phosphate or sugar of the adjacent site in the $5'$ direction are denoted by P($5'$) or S($5'$) respectively. Similarly, the phosphate or sugar in the $3'$ direction are denoted by P($3'$) or S($3'$) respectively.

Bond	r^0 (Å)	Bend	θ^0 (°)	Torsion	ϕ^0 (°)	σ_ϕ
P($5'$)−S	3.899	S−P−S	94.49	($5'$)P−S−P−S($3'$)	−154.79	0.30
S−P($3'$)	3.559	P−S−P	120.15	($5'$)S−P−S−P($3'$)	−179.17	0.30
S−A	4.670	P−S−A	103.53			
S−T	4.189	P−S−T	92.06			
S−G	4.829	P−S−G	107.40			
S−C	3.844	P−S−C	103.79			
		A−S−P	112.07			
		T−S−P	116.68			
		G−S−P	110.12			
		C−S−P	110.33			

Non-bonded potentials

The non-bonded potential $E_{non-bonded}^{DNA}$ is given by,

$$E_{non-bonded}^{DNA} = E_{ev}^{DNA} + E_{elec}^{DNA} + E_{bstk}^{DNA} + E_{bp}^{DNA} + E_{cstk}^{DNA} \tag{S17}$$

where E_{ev}^{DNA} is the potential energy for excluded volume interactions, E_{elec}^{DNA} is the the potential energy for electrostatic interactions, E_{bstk}^{DNA} denotes intra-strand base-stacking interactions, E_{bp}^{DNA} and E_{cstk}^{DNA} are the inter-strand base-pairing and cross-stacking interactions.

Excluded volume interactions between sites i and j are modelled through a purely repulsive potential, given by

$$E_{ev}^{DNA} = \sum_{i<j} \begin{cases} \epsilon_r\left[\left(\frac{\sigma_{ij}}{r_{ij}}\right)^{12} - 2\left(\frac{\sigma_{ij}}{r_{ij}}\right)^{6}\right] + \epsilon_r & r_{ij} < r_c \\ 0 & r_{ij} \geq r_c \end{cases} \tag{S18}$$

where $\epsilon_r = 1.0$ kJ/mol, σ_{ij} is the sum of the radii of sites i and j, r_{ij} is the distance between them and r_c denotes the cutoff distance which is always set to equal to σ_{ij}. Note that, this potential only applies between sites that do not take part in any bonded interactions, base-pairing or base-stacking interactions within the cutoff distance r_c.

Electrostatic interactions are modelled using Debye-Hückel potential given in Eq. S6. Only phosphate beads, which are assigned -0.6 charge, contribute to this potential; the sugar and base atoms are neutral. In the DNA model, the net charge of each phosphate bead is increased from -1.0 to -0.6 in order to take into account the effect of counter-ion condensation. The charged beads participates in the electrostatic interactions between all intra-strand phosphates, which are not from neighbouring nucleotides and all other inter-strand phosphates.

The base-base interactions are introduced through angle-dependent potentials, which can be divided into three components: base-stacking (E^{DNA}_{bstk}), base-pairing (E^{DNA}_{bp}) and cross-stacking (E^{DNA}_{cstk}) interactions. All three interactions rely on a Morse potential of the form

$$U_{Morse}(\epsilon_{ij}, \alpha_{ij}, r_{ij}) = \epsilon_{ij}(1 - e^{(-\alpha_{ij}(r_{ij}-r^0_{ij}))})^2 - \epsilon_{ij} \tag{S19}$$

which can be decomposed into a repulsive component

$$U^{rep}_{Morse}(\epsilon_{ij}, \alpha_{ij}, r_{ij}) = \begin{cases} \epsilon_{ij}(1 - e^{(-\alpha_{ij}(r_{ij}-r^0_{ij}))})^2 & r_{ij} < r^0_{ij} \\ 0 & r_{ij} \geq r^0_{ij} \end{cases} \tag{S20}$$

and an attractive component

$$U^{attr}_{Morse}(\epsilon_{ij}, \alpha_{ij}, r_{ij}) = \begin{cases} -\epsilon_{ij} & r_{ij} < r^0_{ij} \\ \epsilon_{ij}(1 - e^{(-\alpha_{ij}(r_{ij}-r^0_{ij}))})^2 - \epsilon_{ij} & r_{ij} \geq r^0_{ij} \end{cases} \tag{S21}$$

Here, ϵ_{ij} denotes the well depth of attraction between sites i and j, α_{ij} is a parameter used to manage the range of attraction and r^0_{ij} is the equilibrium distance between the interacting sites.

A modulating function f is also incorporated to the angles of interaction in the base-stacking, base-pairing and cross-stacking interactions to smoothly scale the interactions between non-hydrogen bonded and hydrogen bonded base pairs. The form of the modulating function f is given by,

$$f(K, \Delta\theta) = \begin{cases} 1 & -\frac{\pi}{2K} < \Delta\theta < \frac{\pi}{2K} \\ 1 - cos^2(K\Delta\theta) & -\frac{\pi}{K} < \Delta\theta < -\frac{\pi}{2K} \quad \text{or} \quad \frac{\pi}{2K} < \Delta\theta < \frac{\pi}{K} \\ 0 & \Delta\theta < -\frac{\pi}{K} \quad \text{or} \quad \Delta\theta > \frac{\pi}{K} \end{cases} \tag{S22}$$

where the modulating constant K depends on the type of interactions. With these definitions, we can fully write the potential energy function for base-stacking interactions as

$$E^{DNA}_{bstk} = \sum^{n_{bstk}} \begin{cases} U^{rep}_{Morse}(\epsilon_{ij}, \alpha_{BS}, r_{ij}) + f(K_{BS}, \Delta\theta_{BSij})U^{attr}_{Morse}(\epsilon_{ij}, \alpha_{BS}, r_{ij}) & r_{ij} < r^0_{ij} \\ f(K_{BS}, \Delta\theta_{BSij})U^{attr}_{Morse}(\epsilon_{ij}, \alpha_{BS}, r_{ij}) & r_{ij} \geq r^0_{ij} \end{cases} \tag{S23}$$

where θ_{BS} is the angle between the vector connecting sugar and base in the $5'$ direction and the vector joining the two base atoms in the $3'$ direction.

The potential energy function for base-pairing is given by

$$E_{bp}^{DNA} = \sum^{n_{bp}} \begin{cases} U_{Morse}^{rep}(\epsilon_{ij}, \alpha_{BP}, r_{ij}) + \frac{1}{2}(1 + cos(\Delta\phi_1))f(K_{BP}, \Delta\theta_{1ij})f(K_{BP}, \Delta\theta_{2ij})U_{Morse}^{attr}(\epsilon_{ij}, \alpha_{BP}, r_{ij}) \\ \hfill r_{ij} < r_{ij}^0 \\ \frac{1}{2}(1 + cos(\Delta\phi_1))f(K_{BP}, \Delta\theta_{1ij})f(K_{BP}, \Delta\theta_{2ij})U_{Morse}^{attr}(\epsilon_{ij}, \alpha_{BP}, r_{ij}) \qquad r_{ij} \geq r_{ij}^0 \end{cases} \tag{S24}$$

In this case, θ_1 is defined as the angle between the sense-strand sugar, base and the anti-sense base, while θ_2 is the same angle but on the opposite strand of DNA and ϕ_1 is the torsion angle between the angles θ_1 and θ_2.

The cross-stacking interaction is given by

$$E_{cstk}^{DNA} = \sum^{n_{cstk}} f(K_{BP}, \Delta\theta_{3ij})f(K_{CS}, \Delta\theta_{CSij})U_{Morse}^{attr}(\epsilon_{ij}, \alpha_{CS}, r_{ij}) \tag{S25}$$

where θ_3 is the angle between the vectors connecting the sugars to the bases in a W-C base pair and θ_{CS} is the vector connecting the sugar to the base, and the vector connecting the base on the anti-sense strand in the $5'$ to the base in the $3'$ direction on the sense strand.

For completeness, we use the following parameters for the DNA model.

Supplementary Table S3: Different parameters used in 3SPN.2C DNA force field for non-bonded interactions.

Parameter	Value
K_{BS}	6.0
K_{BP}	12.0
K_{CS}	8.0
α_{BS}	3.0
α_{BP}	2.0
α_{CS}	4.0
σ_{AT}	5.941 Å
σ_{GC}	5.518 Å
ϵ_{AT}	14.41 kJ/mol
ϵ_{GC}	18.24 kJ/mol

Supplementary Table S4: The mass (m) and excluded volume radius (r) of each DNA site.

	m (amu)	r (Å)
Phosphate (P)	94.97	2.25
Sugar (S)	83.11	3.20
Adenine (A)	134.1	2.70
Thymine (T)	125.1	3.55
Cytosine (C)	110.1	3.20
Guanine (G)	150.1	2.45

Supplementary Table S5: Reference angles that are used to modulate E_{bp}^{DNA} and E_{cstk}^{DNA}. Here, i and j denotes the identity of the base sites that are used to define the vector r_{ij}.

		Base j								
		ϕ_1^0 (°)					θ_1^0 (°)			
		A	T	G	C		A	T	G	C
	A	...	−38.35	...	...	A	...	156.54	...	...
	T	−38.35	...	...	...	T	135.78	...	...	...
	G	...	...	...	−45.81	G	...	...	...	154.62
	C	...	...	−45.81	...	C	...	...	152.74	...
Base i		θ_2^0 (°)					θ_3^0 (°)			
		A	T	G	C		A	T	G	C
	A	...	135.78	...	...	A	...	116.09	...	...
	T	156.54	...	...	...	T	116.09	...	...	...
	G	...	...	...	152.74	G	...	...	...	131.78
	C	...	...	154.62	...	C	...	...	131.78	...

Supplementary Table S6: Values of strengths ϵ_{ij} for (a) base stacking and (b-c) cross stacking energies in the DNA model. $\uparrow$ and $\downarrow$ denotes the sense and anti-sense strands respectively.

				Base $_{3'}\uparrow$ ϵ_{ij} (kJ/mol)		
(a)			A	T	G	C
	Base $^{5'}\uparrow$	A	13.82	15.05	13.32	15.82
		T	9.15	12.44	9.58	13.11
		G	13.76	14.59	14.77	15.17
		C	9.25	12.42	8.83	14.01
				Base $\downarrow_{5'}$ ϵ_{ij} (kJ/mol)		
(b)			A	T	G	C
	Base $^{5'}\uparrow$	A	1.882	2.388	2.439	1.680
		T	2.388	1.882	2.187	2.566
		G	2.439	2.187	3.250	0.972
		C	1.680	2.566	0.972	4.135
				Base $_{3'}\uparrow$ ϵ_{ij} (kJ/mol)		
(c)			A	T	G	C
	Base $\downarrow^{3'}$	A	1.882	2.388	2.566	2.187
		T	2.388	1.882	1.680	2.439
		G	2.566	1.680	4.135	0.972
		C	2.187	2.439	0.972	3.250

Supplementary Table S7: Values of equilibrium distances and equilibrium angles for (a) base stacking and (b-c) cross stacking energies in the DNA model. $\uparrow$ and $\downarrow$ denotes the sense and anti-sense strands respectively.

					Base $_{3'}\uparrow$					
			σ_{ij} (Å)				θ^0_{BS} (°)			
(a)			A	T	G	C		A	T	G	C
	Base $^{5'}\uparrow$	A	3.716	3.675	3.827	3.975	A	101.15	85.94	105.26	90.26
		T	4.238	3.984	4.416	4.468	T	101.59	89.50	104.31	90.82
		G	3.576	3.598	3.664	3.822	G	100.89	84.83	105.48	90.18
		C	3.859	3.586	4.030	3.957	C	115.95	101.51	119.32	104.49
					Base $\downarrow_{5'}$					
			σ_{ij} (Å)				θ^0_{CS} (°)			
(b)			A	T	G	C		A	T	G	C
	Base $^{5'}\uparrow$	A	6.208	6.876	6.072	6.941	A	154.38	159.10	152.46	157.58
		T	6.876	7.480	6.771	7.640	T	147.10	153.79	144.44	148.59
		G	6.072	6.771	5.921	6.792	G	154.69	157.83	153.43	158.60
		C	6.941	7.640	6.792	7.698	C	160.37	164.45	158.62	162.73
					Base $_{3'}\uparrow$					
			σ_{ij} (Å)				θ^0_{CS} (°)			
(c)			A	T	G	C		A	T	G	C
	Base $\downarrow^{3'}$	A	5.435	6.295	5.183	5.965	A	116.88	121.74	114.23	114.58
		T	6.295	7.195	6.028	6.868	T	109.42	112.95	107.32	106.41
		G	5.183	6.028	4.934	5.684	G	119.34	124.72	116.51	117.49
		C	5.965	6.868	5.684	6.453	C	122.10	125.80	120.00	119.67

Protein−DNA interactions

In our model, we incorporated the following two potential energies to study the non-specific interactions between protein and DNA at the molecular level: (i) the electrostatic interactions between negatively charged phosphate beads and charged amino acids (Arg, Lys, Glu, Asp), and (ii) the repulsive excluded volume interactions between protein residues and DNA beads. Therefore,

$$E_{pot}^{protein-DNA} = E_{elec}^{protein-DNA} + E_{ev}^{protein-DNA} \tag{S26}$$

The electrostatic interactions between charged residues of protein and DNA are modeled using Debye-Hückel potential as given in Eq. S6. Since the phosphate beads are assigned a negative charge of 0.6 in the DNA model, the effective charge of interactions between protein and DNA sites is scaled by a factor of 1.67 in order to bring the local charge of phosphate beads back to −1, as used in the previous work (4).

The excluded volume interactions between protein and DNA beads are modelled by a purely repulsive potential, given by

$$E_{ev}^{protein-DNA} = \sum_{|i-j|>3} \epsilon_{ij} \left(\frac{\sigma_{ij}}{r_{ij}}\right)^{12} \tag{S27}$$

where $\epsilon_{ij} = 1.0$ kJ/mol, σ_{ij} is the average site diameter between sites i and j and r_{ij} is the separation distance between them.

Thus, the total potential energy of the protein−DNA system becomes,

$$E_{pot} = E_{pot}^{protein} + E_{pot}^{DNA} + E_{pot}^{protein-DNA} \tag{S28}$$

Sequence of the 336-bp DNA minicircle

The sequence of a 336-bp DNA minicircle used in our study is the same considered in the previous study combining all-atom simulations and cryo-electron tomography (cryo-ET) experiments (5, 6). The sequence of the 336-bp DNA minicircle is given by:

```
            10         20         30         40         50         60         70
5'–TTTATACTAA CTTGAGCGAA ACGGGAAGGG TTTTCACCGA TATCACCGAA ACGCGCGAGG CAGCTGTATG
            80         90        100        110        120        130        140
   GCATGAAAGA GTTCTTCCCG GAAAACGCGG TGGAATATTT CGTTTCCTAC TACGACTACT ATCAGCCGGA
           150        160        170        180        190        200        210
   AGCCTATGTA CCGAGTTCCG ACACTTTCAT TGAGAAAGAT GCCTCAGCTC TGTTACAGGT CACTAATACC
           220        230        240        250        260        270        280
   ATCTAAGTAG TTGATTCATA GTGACTGCAT ATGTTGTGTT TTACAGTATT ATGTAGTCTG TTTTTTATGC
           290        300        310        320        330
   AAAATCTAAT TTAATATATT GATATTTATA TCATTTTACG TTTCTCGTTC AGCTTT – 3'
```

Definition of Lk, ΔLk and σ

The total number of double-helical turns in a closed circular DNA molecule is defined by the linking number, Lk. For a given length of DNA, Lk is given by the ratio of the total number of DNA base pairs (N_{bp}) and the number of base pairs per helical turn (h), i.e., $Lk = N_{bp}/h$, where $h = 10.5$ for ideal B-DNA geometry. Any change in the Lk value from its relaxed value (Lk_0) causes supercoiling in DNA. The corresponding change in the linking number relative to Lk_0 is described by the term $\Delta Lk = Lk - Lk_0$. The ΔLk is commonly normalised to the size of the circle to give the superhelical density σ, defined by $\sigma = \frac{\Delta Lk}{Lk_0}$.

Criteria for sliding, hopping, 3D-diffusion and intersegmental transfer

We extensively analysed the structural details of protein diffusing on DNA to various search modes, namely sliding, hopping, 3D diffusion, and intersegmental transfer based on the following criteria. We monitored the distance (R) between center of mass of the recognition region of protein and the closest DNA base pair from it (see Figure 2C, upper panel in the main text). A snapshot is considered to be in sliding mode if the recognition region is at a distance of less than 15 Å from DNA ($R \leq 15$ Å). For a multi-domain protein featuring more than one recognition regions, a sliding conformation is further classified into two modes. (i) Pure sliding − when both the recognition regions satisfy the condition $R \leq 15$ Å, and (ii) if only one recognition region satisfies the condition but the other remain dissociated from DNA surface, we consider it a partially dissociated state. Presence of such distinct sliding modes have already been identified (7, 8).

The protein is considered to perform hopping if any one of its recognition region satisfies the condition 15 Å $< R \leq 25$ Å. If both the recognition regions are found at a distance greater than 25 Å from the DNA (i.e., $R > 25$ Å), the protein is considered to be in 3D diffusion mode.

To figure out the snapshot when protein is involved in intersegmental transfer between two distant DNA sites, we monitored the binding position of both the recognition regions of the protein on DNA (Figure 2C, lower panel in the main text). The binding position on DNA is obtained by identifying the index of the closest DNA base pair from the center of mass of the respective recognition region of the protein while performing 1D motion on the DNA. If the two base pair indices are sequentially more than a cut-off value of N_c bp (we choose $N_c = 20$ bp), we considered that the protein is engaged in performing intersegmental jumps between the two DNA sites.

Calculation of rotation-coupled sliding of Fis protein

To capture the rotation-coupled sliding of a protein on DNA, we monitored the rotational angle (θ) and the transversal displacement (along the DNA contour) of the center of mass of the recognition region of the diffusing protein. The rotational angle (in degrees) around the DNA is calculated by $\theta = \tan^{-1}(\frac{y}{x})$, where x and y are the corresponding coordinates of the center of mass of the recognition region of the protein molecule.

Calculation of diffusion coefficients for 1D movement of protein

To obtain the diffusion coefficients (D_1) for the 1D movement of the protein along the DNA, we measured the mean square displacement (MSD) of the parameter b defined below. MSD is derived via the equation (9) :

$$MSD(\tau) = \sum_{i=1}^{t-\tau} \frac{(b_{i+\tau} - b_i)^2}{t-\tau} = 2dD_1\tau \tag{S29}$$

where t is the number of time steps measured, τ denotes the measurement window ranging from 1 to t, and b is the number of new DNA sites (base-pair equivalent) that are visited/probed by the protein during 1D diffusion (i.e., when the protein is involved in the direct base readout of local DNA sequence). Any new DNA site is chosen by identifying the closest DNA site from the center of mass of the protein, which was not scanned earlier by the protein. The $MSD(\tau)$ at τ below a cutoff τ_c will be linear with a slope of $2dD_1$, where d is the dimensionality of diffusion. Finally, D_1 is obtained from the slope of MSD of the parameter b.

Calculation of diffusivity of juxtaposition site

To calculate the diffusivity of the juxtaposition site under different ΔLk values, we first measured the mean square displacement (MSD) of the beginning position of the juxtaposition site (defined in the previous section) via the equation (10) :

$$MSD(\tau) = \sum_{i=1}^{t-\tau} \frac{(j_{beg}(i+\tau) - j_{beg}(i))^2}{t-\tau} \tag{S30}$$

where j_{beg} is the beginning position of the juxtaposition site, t is the number of time steps measured, and τ denotes the measurement window ranging from 1 to t. The diffusion coefficient D_1^{jp} is obtained from the slope of the linear behavior of MSD using the relation $MSD = 2D_1^{jp}\tau$.

Calculation of helical twist

The twist angle is defined as the average angle between the nearest neighbour vectors $\vec{l_i}$ and $\vec{l}_{i+1}$ of the base pair and is calculated by the following equation (11) :

$$Tw = \frac{1}{N_{bp}-1} \sum_{i=1}^{N_{bp}} cos^{-1}\left(\frac{\vec{l_i} \,.\, \vec{l}_{i+1}}{|\vec{l_i}||\vec{l}_{i+1}|}\right) \tag{S31}$$

where N_{bp} is the total number of base pairs and $\vec{l_i}$ is the vector connecting the two bases in the ith base pair.

Calculation of writhe

We follow the methodology used previously for the calculation of writhe (12, 13). If $\vec{r}(s)$ represents the helical axis of a closed DNA minicircle of length L interpolating the center of mass of base atoms, then writhe (Wr) can be

calculated from the Gauss′ Integral formula:

$$Wr = \frac{1}{4\pi}\oint_0^L \oint_0^L \left(\frac{d\vec{r}(s)/ds}{|d\vec{r}(s)/ds|} \times \frac{d\vec{r}(s')/ds'}{|d\vec{r}(s')/ds'|}\right) \cdot \frac{\left(\vec{r}(s) - \vec{r}(s')\right)}{|\vec{r}(s) - \vec{r}(s')|^3} ds' ds \tag{S32}$$

If $\{\vec{r}_i\}$ represent a discrete set of representative coordinates of an N_{bp} base pairs minicircle, then the above Gauss′ Integral can be approximated as a summation:

$$Wr \approx \frac{1}{2\pi}\sum_{i=1}^{N_{bp}}\sum_{j\geq i}^{N_{bp}} \left(\frac{\vec{r}_{i+1} - \vec{r}_i}{|\vec{r}_{i+1} - \vec{r}_i|} \times \frac{\vec{r}_{j+1} - \vec{r}_j}{|\vec{r}_{j+1} - \vec{r}_j|}\right) \cdot \frac{\left(\vec{r}_i - \vec{r}_j\right)}{|\vec{r}_i - \vec{r}_j|^3} \tag{S33}$$

Note that even though DNA structures are classified by the number of helical turns, their signs can also be directly found from the calculation of writhe value. Thus, the sign of withe value gives an alternative way to distinguish the negative/positive supercoiled DNA (see Supplementary Figure S9).

Radius of gyration

The radius of gyration is defined by the root mean square distance of the atoms about the centre of mass $\vec{r}_{CM}$:

$$R_g = \sqrt{\frac{1}{N}\sum_{i=1}^{N}\left(\vec{r}_i - \vec{r}_{CM}\right)^2} \tag{S34}$$

The summation is performed over all the N beads with coordinates $\{\vec{r}_i\}$ at each MD time step.

Definition of DNA curvature at the protein binding site

To define the curvature of DNA at the protein binding site, we first identified the DNA index i which is the closest to the center of mass of the protein when the protein diffuses one dimensionally along the DNA. The curvature k is then defined as the inverse of the radius of a circle passing through three DNA sites at indices $i-5$, i, and $i+5$.

Kinetic model for finding target sites on supercoiled DNA by proteins

Here we describe our previously developed theoretical framework for finding target sites by proteins on closed circular DNA and on supercoiled DNA with varying number of juxtaposition sites (14). First, we consider a closed circular DNA and a searching protein (Supplementary Figure S14). The DNA molecule comprises of L binding sites, out of which $L-1$ sites are considered as non-specific binding sites (blue circles) and one site is chosen as the target site (red circle) at which the protein can bind specifically. We placed the target position at the Lth site, because all the sites on a closed circular DNA are identical. The protein begins the search process from the solution phase, labeled as state 0. From the solution, the protein can associate to any non-specific site on closed circular DNA with rate k_{on}. In the reverse reaction, the protein can dissociate from any non-specific DNA site into the solution with rate k_{off}. Once the protein lands on any non-specific DNA site, it can slide along the DNA

contour either in forward and backward directions with the diffusion rate u. Here, we adopted the experimentally determined (15, 16) association and dissociation rates of Fis protein as input parameters.

To analyze the dynamic description of target search process on closed circular DNA, we define a function $P_n(t)$ that denotes the probability of the protein to reach the target site on DNA at time t, if at $t = 0$ the protein was at state n (where $n = 1, 2, \ldots, L$ are sites on the DNA and $n = 0$ signifies bulk solution phase). The temporal evolution of $P_n(t)$ can be described by a set of backward master equations,

$$\frac{dP_n(t)}{dt} = u\big[P_{n-1}(t) + P_{n+1}(t)\big] + k_{off}P_0(t) - (2u + k_{off})P_n(t), \text{ for } 2 \le n \le L-2 \tag{S35}$$

The equations for the adjacent sites to the target (L^{th} site) in a closed circular DNA takes the form

$$\frac{dP_1(t)}{dt} = u\big[P_2(t) + P_L(t)\big] + k_{off}P_0(t) - (2u + k_{off})P_1(t) \tag{S36}$$

$$\frac{dP_{L-1}(t)}{dt} = u\big[P_{L-2}(t) + P_L(t)\big] + k_{off}P_0(t) - (2u + k_{off})P_{L-1}(t) \tag{S37}$$

In a similar manner, the time evolution of the solution phase is given by

$$\frac{dP_0(t)}{dt} = \frac{k_{on}}{L}\sum_{n=1}^{L} P_n(t) - k_{on}P_0(t) \tag{S38}$$

Since, protein molecule moves much faster in solution than on DNA, in Eq. S38, we assumed all the DNA sites are equally accessible (with rate k_{on}/L per DNA site) for the searching protein to associate nonspecifically.

The initial condition is set to $P_L(0) = \delta_{L,0}$, which suggests that if at time $t = 0$ the protein was at target site L, the search process finishes immediately.

To solve Eqs. S35−S38, we adopt the Laplace transformation $\widetilde{P_n}(s) = \int_0^\infty e^{-st}P_n(t)dt$ that transforms the equations into simple algebraic forms as follows:

$$(s + 2u + k_{off})\widetilde{P_n}(s) = u\Big[\widetilde{P_{n-1}}(s) + \widetilde{P_{n+1}}(s)\Big] + k_{off}\widetilde{P_0}(s) \tag{S39}$$

$$(s + 2u + k_{off})\widetilde{P_1}(s) = u\big[\widetilde{P_2}(s) + 1\big] + k_{off}\widetilde{P_0}(s) \tag{S40}$$

$$(s + 2u + k_{off})\widetilde{P_{L-1}}(s) = u\big[\widetilde{P_{L-2}}(s) + 1\big] + k_{off}\widetilde{P_0}(s) \tag{S41}$$

$$(s + k_{on})\widetilde{P_0}(s) = \frac{k_{on}}{L}\sum_{n=1}^{L}\widetilde{P_n}(s) \tag{S42}$$

with the initial condition $\widetilde{P_L}(s) = 1$.

Eqs. S39−S42 can be solved simultaneously by assuming a general solution of the form $\widetilde{P_n}(s) = A(s)y(s)^n + B(s)$. This yields

$$\widetilde{P_n}(s) = (1 - B)\frac{y^n + y^{L-n}}{1 + y^L} + B, \text{ for } n > 0 \tag{S43}$$

For $n = 0$, the solution becomes

$$\widetilde{P_0}(s) = \frac{k_{on}(s + k_{off})S_i(s)}{Ls(s + k_{on} + k_{off}) + k_{on}k_{off}S_i(s)} \tag{S44}$$

where parameters y and B are given by

$$y(s) = \frac{(s+2u+k_{off}) - \sqrt{(s+2u+k_{off})^2 - 4u^2}}{2u},$$
$$B = \frac{k_{off}}{s+k_{off}}\widetilde{P_0}(s) \tag{S45}$$

Therefore, the mean first-passage time (MFPT) to locate the target site on closed circular DNA can be found directly from the Laplace transformations

$$T_0 = \int_0^\infty tP_0(t)dt = -\frac{\partial \widetilde{P_0}(s)}{\partial s}|_{s=0} \tag{S46}$$

which yields to,

$$T_0 = \frac{k_{off}L + k_{on}[L - S_i(0)]}{k_{on}k_{off}S_i(0)} \tag{S47}$$

where the auxiliary function for closed circular DNA is given by

$$S_i(s) = \frac{(1+y)(1-y^L)}{(1-y)(1+y^L)} \tag{S48}$$

Next, we consider supercoiled DNA topology in which two sequentially distant DNA sites come spatially close to each other and may promote the transfer of a protein molecule from one DNA site to another by binding transiently to both the sites, a mechanism known as the "intersegmental transfer". If the corresponding transition rate is k_t, intercommunication of this nature between two sites m and m_b (see Supplementary Figure S14B) imposes a condition given by,

$$\frac{dP_m(t)}{dt} = u\big[P_{m-1}(t) + P_{m+1}(t)\big] + k_t P_{m_b}(t) + k_{off}P_0(t) - (2u + k_{off} + k_t)P_m(t) \tag{S49}$$

This equation is similar to the Eq. S35 with the additional second term that takes into account the intersegmental jump between sites m and m_b with rate k_t. The corresponding Laplace transformation results into

$$(s+2u+k_{off}+k_t)\widetilde{P_m}(s) = u\Big[\widetilde{P_{m-1}}(s) + \widetilde{P_{m+1}}(s)\Big] + k_t\widetilde{P_{m_b}}(s) + k_{off}\widetilde{P_0}(s) \tag{S50}$$

Therefore, the average search time for a protein on a supercoiled DNA with one crossover site (Supplementary Figure S14B) can be estimated by solving Eqs. S39−S42 and Eq. S50 simultaneously. The explicit form of the MFPT remains the same as in Eq. S47 with differences in auxiliary function $S_i(s)$. For supercoiled DNA with one crossover site $S_i(s)$ is given by,

$$S_i(s) = \frac{y^m(1+y)(1-y^L)}{(1-y)}.\frac{\theta_1}{\theta_2} \tag{S51}$$

where

$$\theta_1 = y(y^m-1)\{(s+2u+k_{off}+k_t)(y^L - y^m) + k_t(y^{m_b} - y^{L+m-m_b})\} + u\{(1+y^2)(y^L+y^{2m}) - 2y^m(y^L+y^2)\}$$

and

$$\theta_2 = y(y^{2m}-1)\{(s+2u+k_{off}+k_t)(y^{2L} - y^{2m}) + k_t y^m(y^{m_b} - y^{2L-m_b})\} + u\{(1+y^2)(y^{2L}+y^{4m}) - 2y^{2m}(y^{2L}+y^2)\}$$

We note that the target site will be maximum approachable when m_b juxtaposes with the target site L. In this situation, the searching protein directly lands on the target site L as soon it performs an intersegmental jump from the juxtaposition site m. Correspondingly, m_b will be replaced by L in Eq. S50 and therefore changes to

$$(s+2u+k_{off}+k_t)\widetilde{P_m}(s) = u\Big[\widetilde{P_{m-1}}(s) + \widetilde{P_{m+1}}(s)\Big] + k_t + k_{off}\widetilde{P_0}(s) \tag{S52}$$

Solving Eqs. S39−S42 and S52 simultaneously, we find the auxiliary function for supercoiled DNA with one juxtaposition site as

$$S_i(s) = \frac{y^m(1+y)(1-y^L)}{(1-y)}\theta_{1jp} \tag{S53}$$

where

$$\theta_{1jp} = \frac{(s+2u+k_{off}+2k_t)y(y^L-y^m)(y^m-1)+u\{(1+y^2)(y^L+y^{2m})-2y^m(y^L+y^2)\}}{(s+2u+k_{off}+k_t)y(y^{2L}-y^{2m})(y^{2m}-1)+u\{(1+y^2)(y^{2L}+y^{4m})-2y^{2m}(y^{2L}+y^2)\}}$$

Finally, we increase the number of juxtaposition points in supercoiled DNA and consider supercoiled DNA topology with two (j_1 and j_2) and three (j_1, j_2 and j_3) juxtaposition sites (see Supplementary Figure S14C and S14D). As a result, at these juxtaposition points, we have the equations

$$(s+2u+k_{off}+k_t)\widetilde{P_{j_1}}(s) = u\left[\widetilde{P_{j_1-1}}(s)+\widetilde{P_{j_1+1}}(s)\right]+k_t+k_{off}\widetilde{P_0}(s) \tag{S54}$$

$$(s+2u+k_{off}+k_t)\widetilde{P_{j_2}}(s) = u\left[\widetilde{P_{j_2-1}}(s)+\widetilde{P_{j_2+1}}(s)\right]+k_t+k_{off}\widetilde{P_0}(s) \tag{S55}$$

$$(s+2u+k_{off}+k_t)\widetilde{P_{j_3}}(s) = u\left[\widetilde{P_{j_3-1}}(s)+\widetilde{P_{j_3+1}}(s)\right]+k_t+k_{off}\widetilde{P_0}(s) \tag{S56}$$

In a similar manner, the auxiliary function $S_i(s)$ can be found for both supercoiled DNA with two and three juxtaposition points which leads to a very complex expression and therefore below we provide only the expression of the auxiliary function for two juxtaposition sites ($j_1 = m_1$ and $j_2 = m_1 + m_2$) and is given by

$$S_i(s) = \frac{\theta_1^{2jp}}{\theta_2^{2jp}} \tag{S57}$$

where

$\theta_1^{2jp} = (1+y)\big[k_t^2y^2(y^{m_1}-1)(y^{m_2}-1)(y^{2L+m_1}+y^{2L+m_2}+3y^{2(m_1+m_2)}-y^{3(m_1+m_2)}-2y^{L+m_1+m_2}+3y^{2L+m_1+m_2}-2y^{L+2m_1+m_2}-2y^{L+m_1+2m_2}+y^{3m_1+2m_2}+y^{2m_1+3m_2}-2y^{L+2m_1+2m_2}-y^{2L})+y^{m_1}\{k_{off}y(y^L-y^{m_2})(y^{m_2}-1)+u(y-1)(y^{1+L}-y^L-y^{2m_2}-2y^{1+m_2}+2y^{L+m_2}+y^{1+2m_2})\}\{k_{off}y(y^{m_1}-1)(y^{m_2}+1)(y^L-y^{m_1+m_2})+u(y-1)(y^{1+L}-y^L+y^{L+m_1}-y^{1+L+m_1}-y^{L+m_2}+y^{1+L+m_2}-y^{2(m_1+m_2)}-2y^{1+m_1+m_2}+2y^{L+m_1+m_2}-y^{2m_1+m_2}+y^{1+2m_1+m_2}+y^{m_1+2m_2}-y^{1+m_1+2m_2}+y^{1+2m_1+2m_2})\}+k_ty\{k_{off}y(y^{m_1}-1)(y^{m_2}-1)(2y^{2L+m_1}+y^{2L+m_2}+4y^{2(m_1+m_2)}-y^{3(m_1+m_2)}-3y^{L+m_1+m_2}+4y^{2L+m_1+m_2}-3y^{L+2m_1+m_2}-3y^{L+m_1+2m_2}+y^{3m_1+2m_2}+2y^{2m_1+3m_2}-3y^{L+2m_1+2m_2}-y^{2L})+u(y-1)(y^{1+2L}-y^{2L}-2y^{2(L+m_1)}+3y^{2L+m_1}-3y^{1+2L+m_1}+2y^{1+2L+2m_1}-y^{2(L+m_2)}+2y^{2L+m_2}-2y^{1+2L+m_2}+2y^{3(m_1+m_2)}-y^{4(m_1+m_2)}-y^{L+m_1+m_2}+8y^{2(L+m_1+m_2)}+5y^{1+L+m_1+m_2}-2y^{2L+m_1+m_2}-2y^{1+2L+m_1+m_2}-y^{2(2m_1+m_2)}-2y^{2L+2m_1+m_2}+2y^{1+2L+2m_1+m_2}+3y^{L+3m_1+m_2}-3y^{1+L+3m_1+m_2}+y^{1+2L+2m_2}-2y^{2(m_1+2m_2)}+2y^{L+m_1+2m_2}+2y^{1+L+m_1+2m_2}-5y^{2L+m_1+2m_2}+y^{1+2L+m_1+2m_2}-8y^{1+2m_1+2m_2}-y^{3m_1+2m_2}+5y^{1+3m_1+2m_2}-2y^{L+3m_1+2m_2}-2y^{1+L+3m_1+2m_2}+y^{1+4m_1+2m_2}+3y^{L+m_1+3m_2}-3y^{1+L+m_1+3m_2}-2y^{2m_1+3m_2}+2y^{1+2m_1+3m_2}+2y^{1+3m_1+3m_2}+y^{1+L+3m_1+3m_2}+2y^{4m_1+3m_2}-2y^{1+4m_1+3m_2}+2y^{1+2m_1+4m_2}+3y^{3m_1+4m_2}-3y^{1+3m_1+4m_2}+y^{1+4m_1+4m_2}-5y^{L+3m_1+3m_2})\}\big]$

$\theta_2^{2jp} = (y-1)\big[k_{off}^2y^2(y^{2m_1}-1)(y^{2m_2}-1)(y^{2L}-y^{2m_1+2m_2})+k_t^2y^2(y^{2m_1}-1)(y^{2m_2}-1)(y^{2L}-y^{2m_1+2m_2})+k_tuy(y-1)(y^{2L}-3y^{1+2L}-2y^{2(L+m_1)}+2y^{1+2L+2m_1}-3y^{2(L+m_2)}-3y^{4(m_1+m_2)}+4y^{2(L+m_1+m_2)}+y^{2(2m_1+m_2)}+y^{1+2L+2m_2}+2y^{2(m_1+2m_2)}+4y^{1+2m_1+2m_2}-3y^{1+4m_1+2m_2}-2y^{1+2m_1+4m_2}+y^{1+4m_1+4m_2})+u^2(y-1)^2(2y^{2+2L}-2y^{1+2L}-y^{2(L+m_1)}-$

$y^{2(1+L+m_1)}+2y^{1+2L+2m_1}-2y^{2(L+m_2)}-2y^{4(m_1+m_2)}-4y^{2(1+m_1+m_2)}+4y^{2(L+m_1+m_2)}+2y^{2(1+2m_1+m_2)}+2y^{1+2L+2m_2}+y^{2(m_1+2m_2)}-2y^{1+4m_1+2m_2}-2y^{1+2m_1+4m_2}+y^{2+2m_1+4m_2}+2y^{1+4m_1+4m_2})+k_{off}y\{2k_ty(y^{2m_1}-1)(y^{2m_2}-1)(y^{2L}-y^{2m_1+2m_2})+u(y-1)(y^{2L}-3y^{1+2L}-2y^{2(L+m_1)}+2y^{1+2L+2m_1}-3y^{2(L+m_2)}-3y^{4(m_1+m_2)}+4y^{2(L+m_1+m_2)}+y^{2(2m_1+m_2)}+y^{1+2L+2m_2}+2y^{2(m_1+2m_2)}+4y^{1+2m_1+2m_2}-3y^{1+4m_1+2m_2}-2y^{1+2m_1+4m_2}+y^{1+4m_1+4m_2})\}]$

Protocol for kinetic Monte Carlo simulations

Our analytical model described above, however, is limited to a pinned juxtaposition site and does not consider the role of juxtaposition site dynamics as found modulated by torsional behavior of arrays of DNA base-pairs in negatively supercoiled DNA conformations. Therefore, to understand the impact of juxtaposition site dynamics on the target search kinetics of proteins on supercoiled DNA, we incorporated the dynamics of a juxtaposition site on supercoiled DNA into our previously developed kinetic Monte Carlo (MC) simulation scheme (14). Here we describe the MC scheme in detail. First, we generated a 336 base pair supercoiled DNA with one juxtaposition site using self-avoiding random walk on a 3D lattice of size $100 \times 100 \times 100$. Any lattice site which is not occupied by the DNA chain is considered as the bulk solution and initially, we placed a single protein molecule randomly in the solution. From the solution, the protein can associate to any lattice sites on the supercoiled DNA with a total association rate k_{on} (k_{on}/L per DNA site). When the protein is non-specifically (other than the target site placed at Lth position) bound to DNA, it may slide along the DNA contour either in forward or backward direction with rate u or it can dissociate into the solution with the rate k_{off}. If the protein reaches the juxtaposition site m, it can perform an intersegmental jump to the sequentially distant DNA site m_b with rate k_t. The simulation strategy is as follows: At each MC step, we generate a random number, r ($0 \leq r \leq 1$) and multiply it with the sum of the rates S $(= u + k_{on} + k_{off} + k_t)$. If $rS \leq k_{on}$, the diffusing protein can bind to any DNA site. Once the protein is non-specifically bound to DNA, it can dissociate into the solution if the condition $k_{on} < rS \leq (k_{on} + k_{off})$ satisfies, whereas for $(k_{on} + k_{off}) < rS \leq (u + k_{on} + k_{off})$, the protein can perform sliding dynamics to adjacent DNA sites. The intersegmental jumps from the juxtaposition site happen when $(u + k_{on} + k_{off}) < rS \leq (u + k_{on} + k_{off} + k_t)$. In this situation, we modulated the dynamics of the juxtaposition site using the following relation:

$$jp^{new} = jp^{old} + \delta(r_1 - 0.5) \tag{S58}$$

where jp^{new} and jp^{old} are the positions of the juxtaposition site in the present and previous MC steps respectively, and r_1 is another random number between 0 and 1. The parameter δ is responsible for the dynamics of the juxtaposition site. After each MC step, the time is advanced by $1/S$. If the protein molecule reaches its target site on supercoiled DNA either through 3D diffusion or by 1D sliding or by intersegmental jumps or by a combination of all, the simulation ends there and the total search time is saved.

In Supplementary Figure S15A, we present the average target search time for a slow ($\delta = 50$) and fast ($\delta = 70$) juxtaposition dynamics. The choice of these two δ values is such that the relative diffusivity of juxtaposition sites in Monte Carlo simulations matches with the ratio of D_1^{jp} values (~ 1.38 times, see Figure 5C in the main text) for $\Delta Lk = -1$ and $\Delta Lk = +3$ obtained in our MD simulation. The MC result shows that the fast dynamics of

juxtaposition sites reduces the average target search time significantly (20%), supporting our observation from the MD simulations (24%). In Supplementary Figure S15B, we scanned a wide range of δ values and computed the required time to reach the target site using the MC scheme. Our result shows that the target search time decreases steadily with the increase in the juxtaposition site dynamics as revealed from our MD simulations.

Calculation of ruggedness of the chemical potential energy landscape

To calculate the ruggedness of the chemical potential energy landscape from the simulation trajectories, we followed the method provided by Putzel *et al* (17). We take the beginning position of the juxtaposition site as the order parameter and divided the whole simulation box into small cubic cells of dimension 10 Å^3. We then measure the probability (p_i^{cell}) of the cell i to be occupied by the diffusing site (juxtaposition beginning site). While diffusing the juxtaposition beginning site from cell i to cell j, the corresponding change in the free energy is given by $-k_BT \, . \ln\big(p_i^{cell}/p_j^{cell}\big)$, which is the excess chemical potential. Therefore, by calculating the standard deviation of the average of each cell's potential gives a measure of ruggedness of the chemical potential energy landscape, given by (in units of k_BT)

$$\sigma(\mu) = \sqrt{\frac{1}{N_{cells}} \sum_i \Big(\ln\big(p_i^{cell}\big) - \overline{\ln\big(p_i^{cell}\big)}\Big)^2} \tag{S59}$$

where N_{cells} is the number of cubic cells.

Supplementary Figures

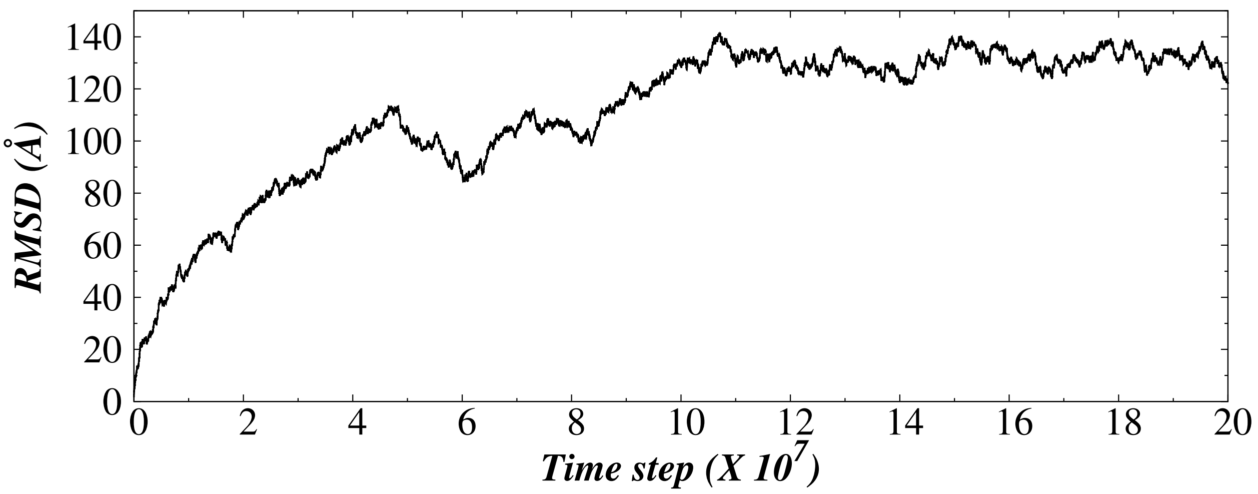

Supplementary Figure S1: Root-mean-square deviation (RMSD) of a 336 bp minicircle DNA as a function of simulation time step for $\Delta Lk = +3$.

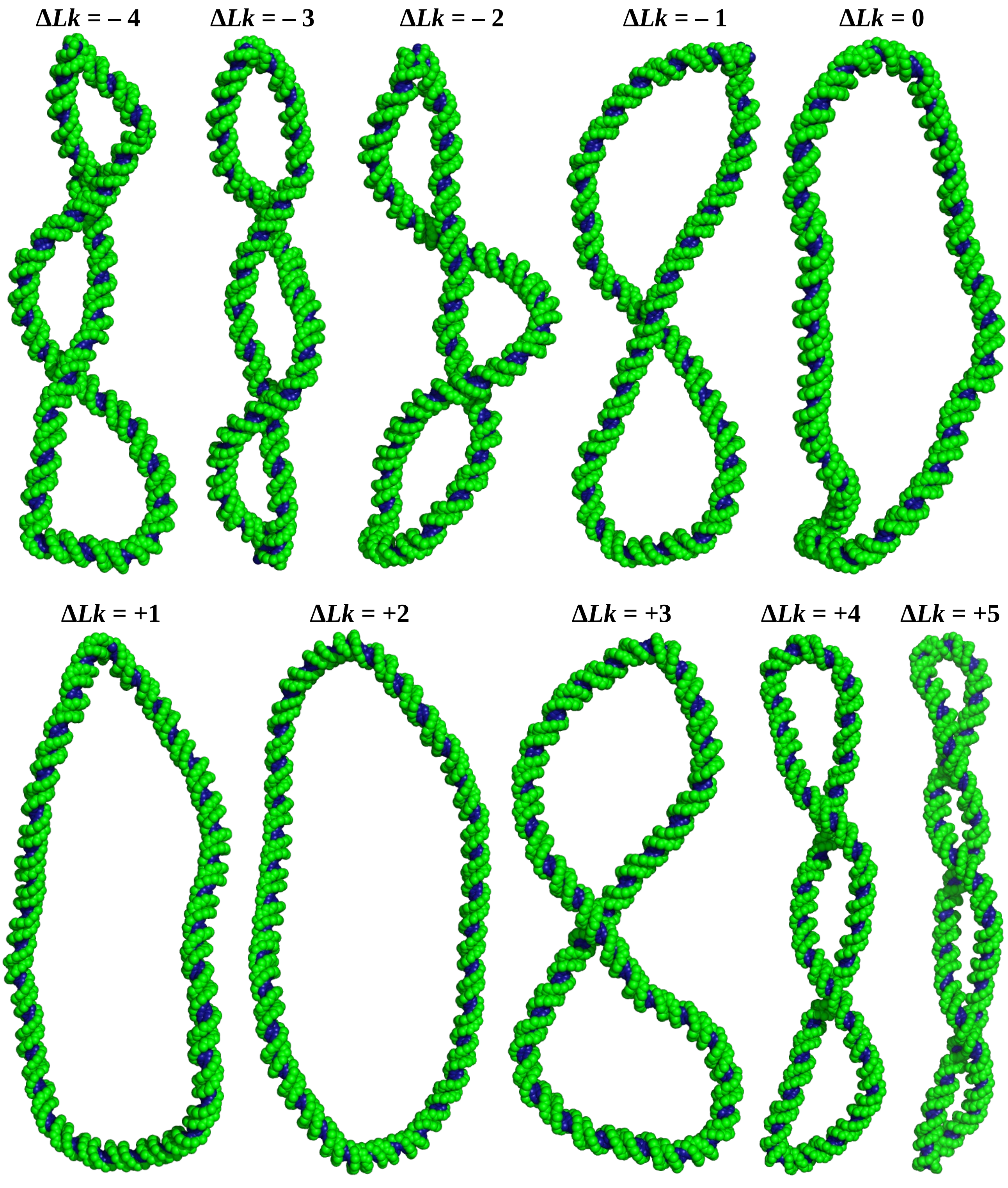

Supplementary Figure S2: Initial starting structure of supercoiled DNA of length 336 bp at each ΔLk value. These structures are used to study the diffusion of protein on plectonemic supercoiled DNA.

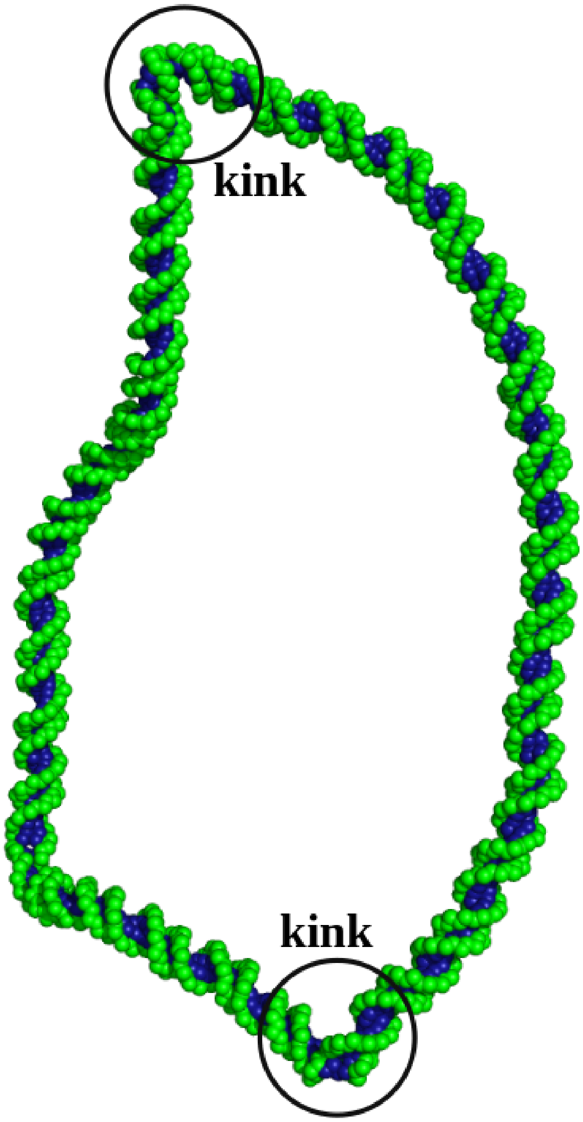

Supplementary Figure S3: Snapshot of a 336 bp minicircle DNA with kinks. Black circles highlight the kinks appeared on the DNA.

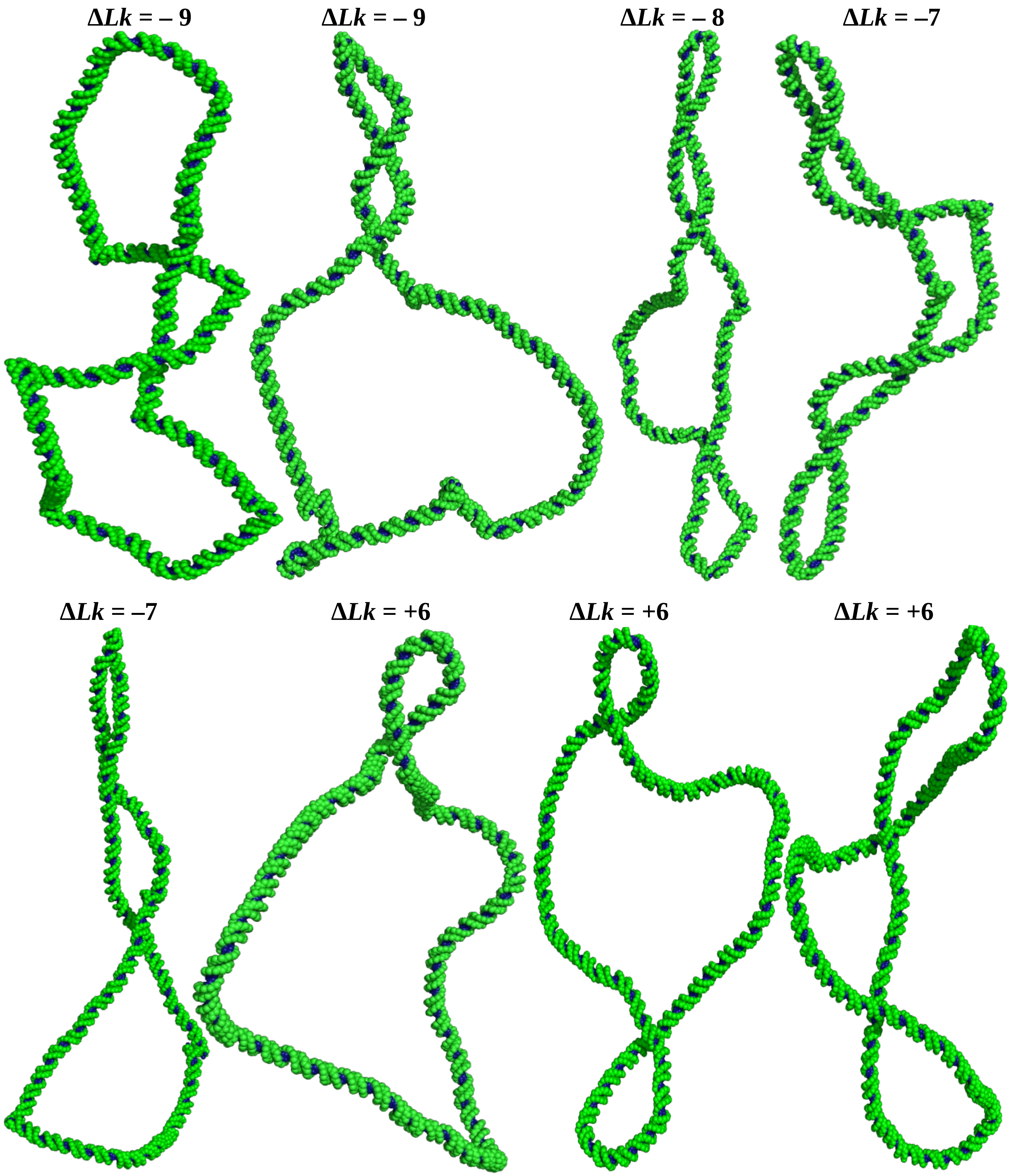

Supplementary Figure S4: Different structures of supercoiled DNA obtained from our coarse-grained simulation of double-length (672 bp) minicircle with negative ($\Delta Lk = -7, -8, -9$) and positive ($\Delta Lk = +6$) supercoiling.

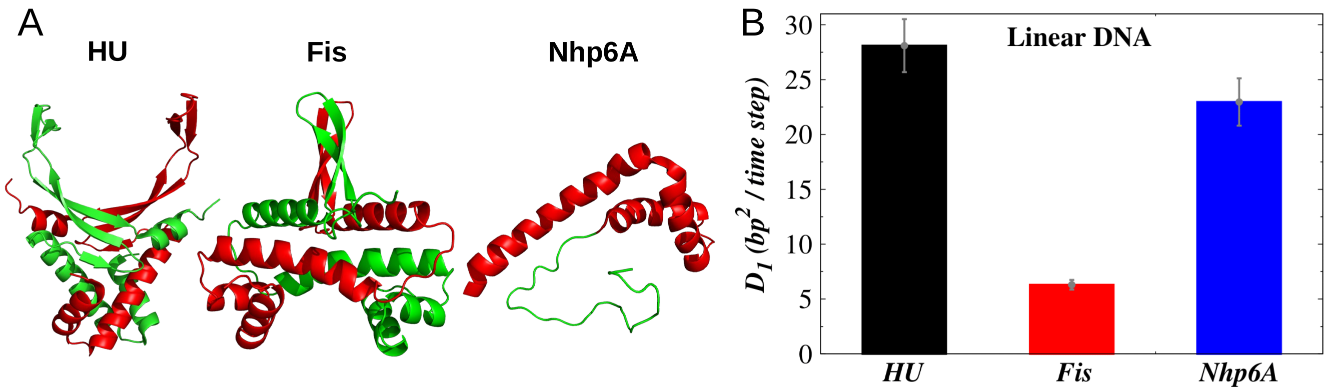

Supplementary Figure S5: (A) Structures of three architectural proteins HU, Fis and Nhp6A. PDB codes used for HU, Fis and Nhp6A are 1P71, 3IV5 and 1J5N, respectively. (B) One dimensional diffusion coefficient (D_1) of three proteins HU, Fis and Nhp6A on linear DNA.

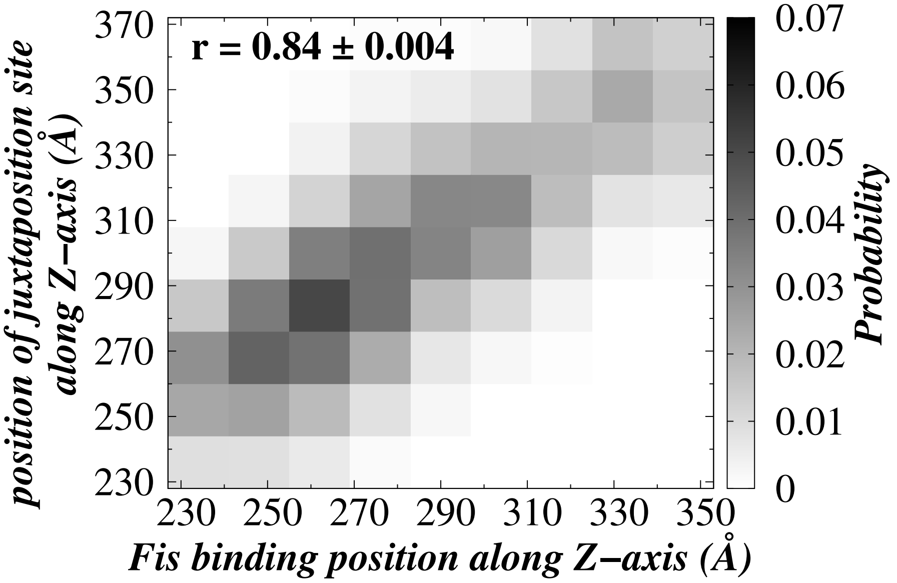

Supplementary Figure S6: Probability histogram of the correlated movement (denoted by the correlation coefficient r) of juxtaposition site and the Fis protein along the Z-direction.

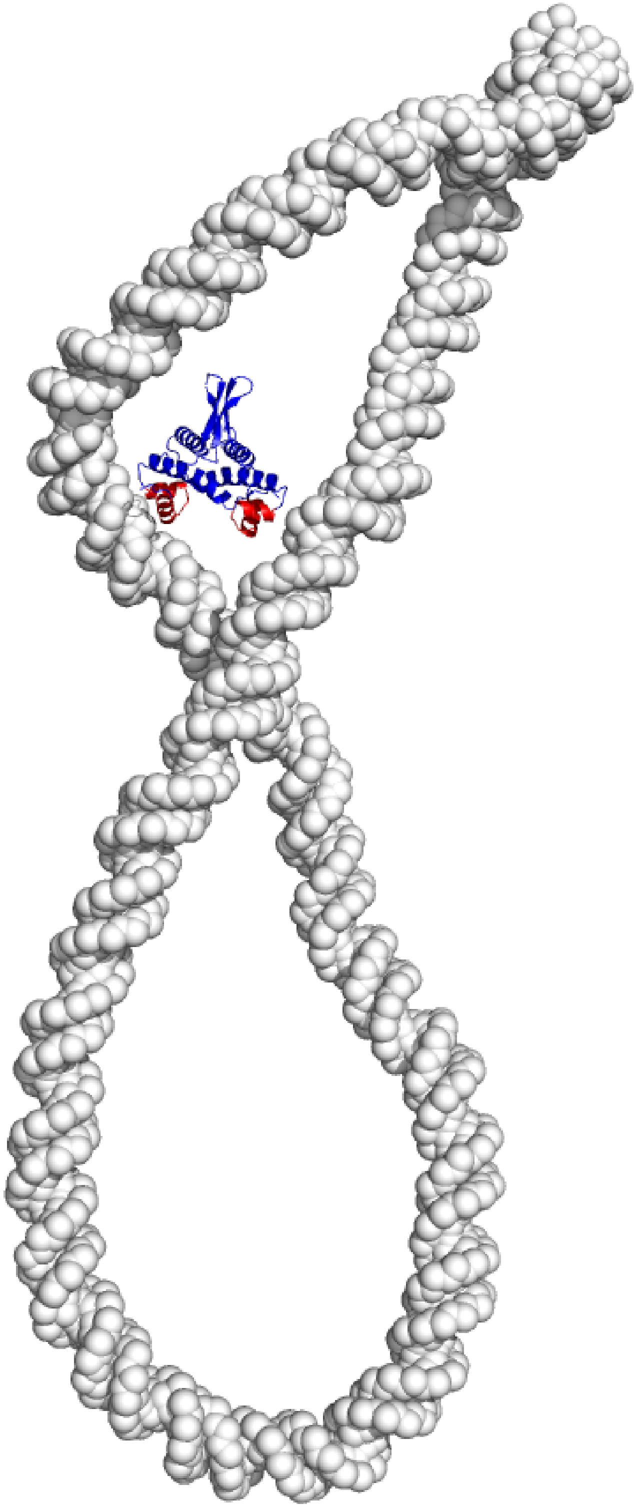

Supplementary Figure S7: Snapshot of transient bridged complex of Fis protein with two sequentially distant DNA sites through intersegmental transfer at the juxtaposition site.

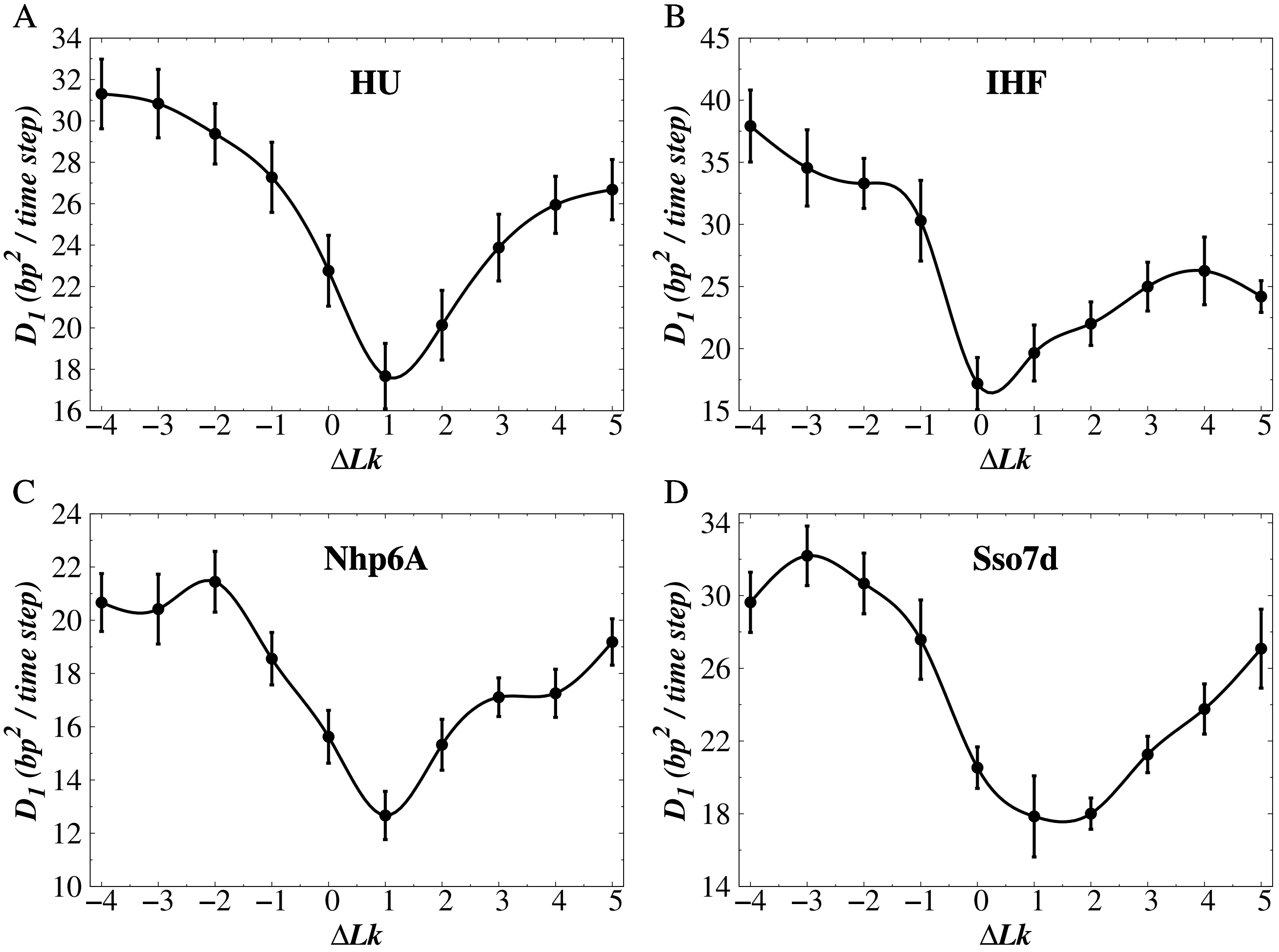

Supplementary Figure S8: 1D diffusion coefficient D_1 as a function of change in linking number ΔLk for (A) HU protein, (B) IHF (Integration Host Factor) protein, (C) Nhp6A protein and (D) Sso7d protein.

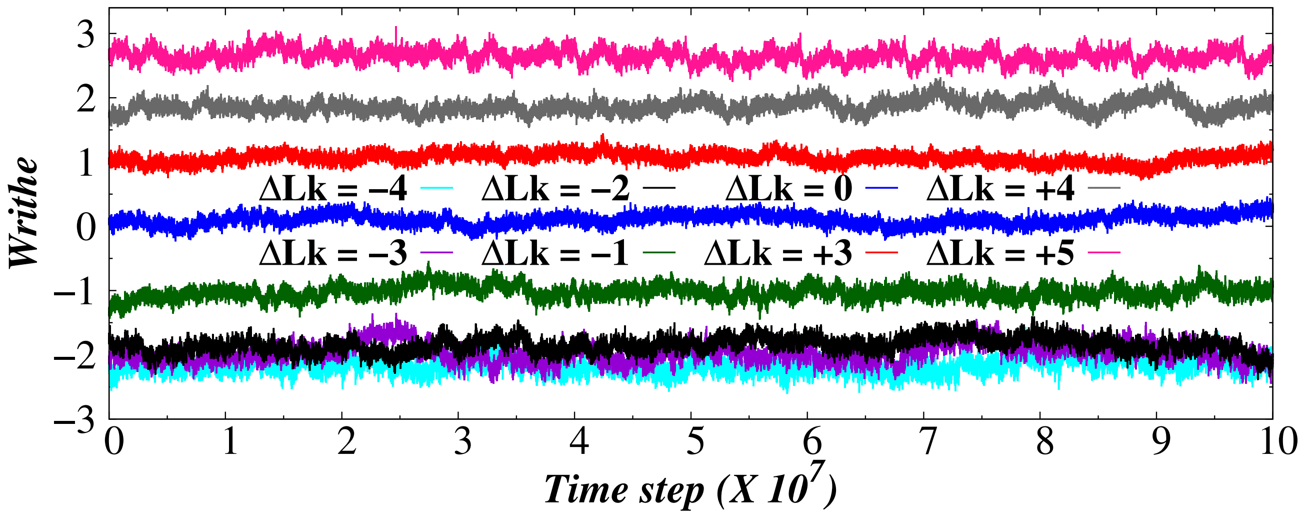

Supplementary Figure S9: Writhe of a 336 bp supercoiled DNA as a function of simulation time step for $\Delta Lk = -4$ (cyan), -3 (violet), -2 (black), -1 (green), 0 (blue), $+3$ (red), $+4$ (grey), $+5$ (pink).

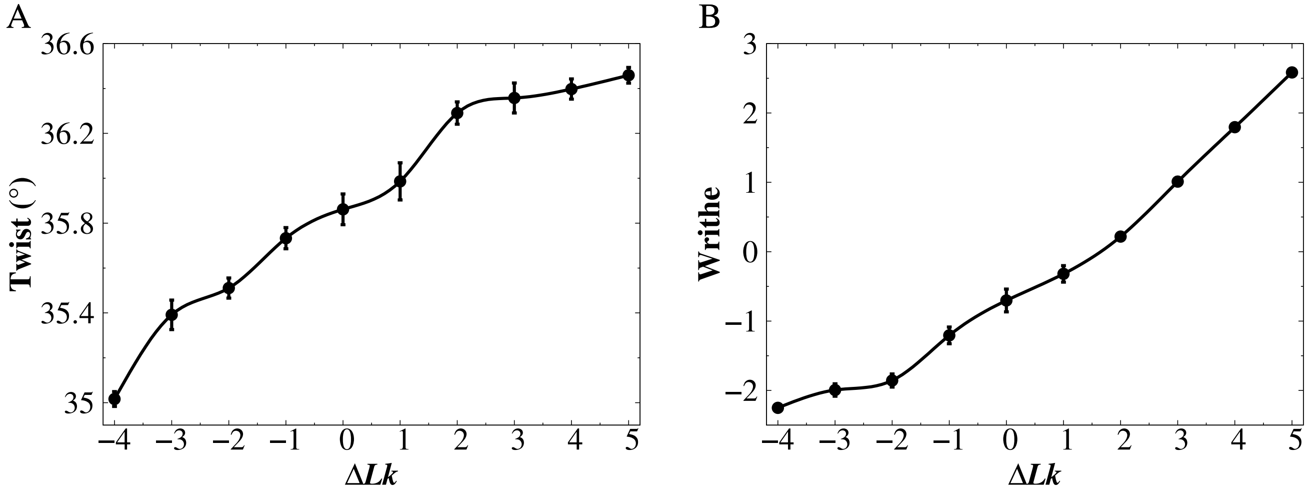

Supplementary Figure S10: Variation in (A) average twist per base pair and (B) writhe as function of change in linking number ΔLk.

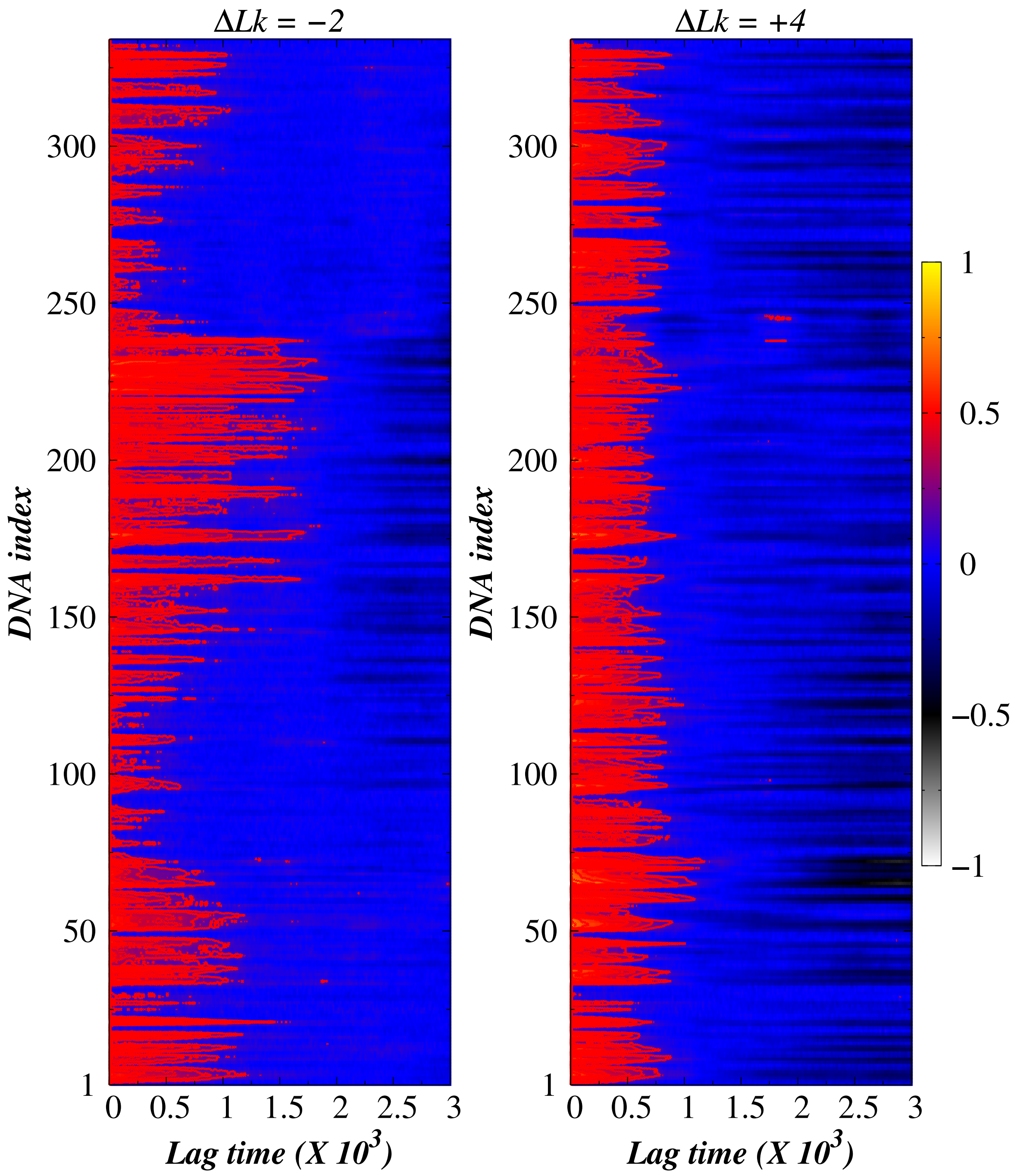

Supplementary Figure S11: DNA twist propagation along arrays of DNA base pairs. Autocorrelation of $C(t,i)$ landscape for arrays of DNA base pairs with $\Delta Lk = -2$ and $+4$. The index of each DNA base in the array is marked on the y-axis.

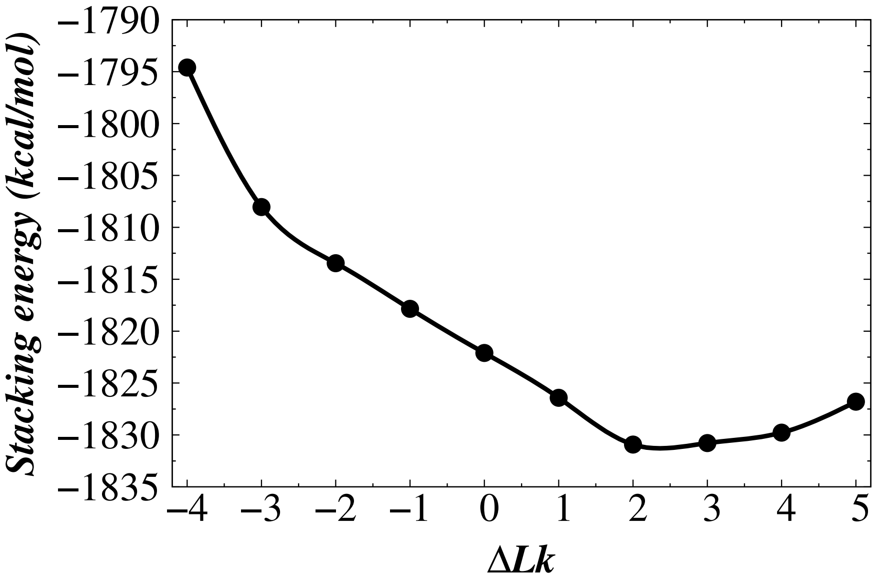

Supplementary Figure S12: DNA stacking energies as a function of change in linking number ΔLk.

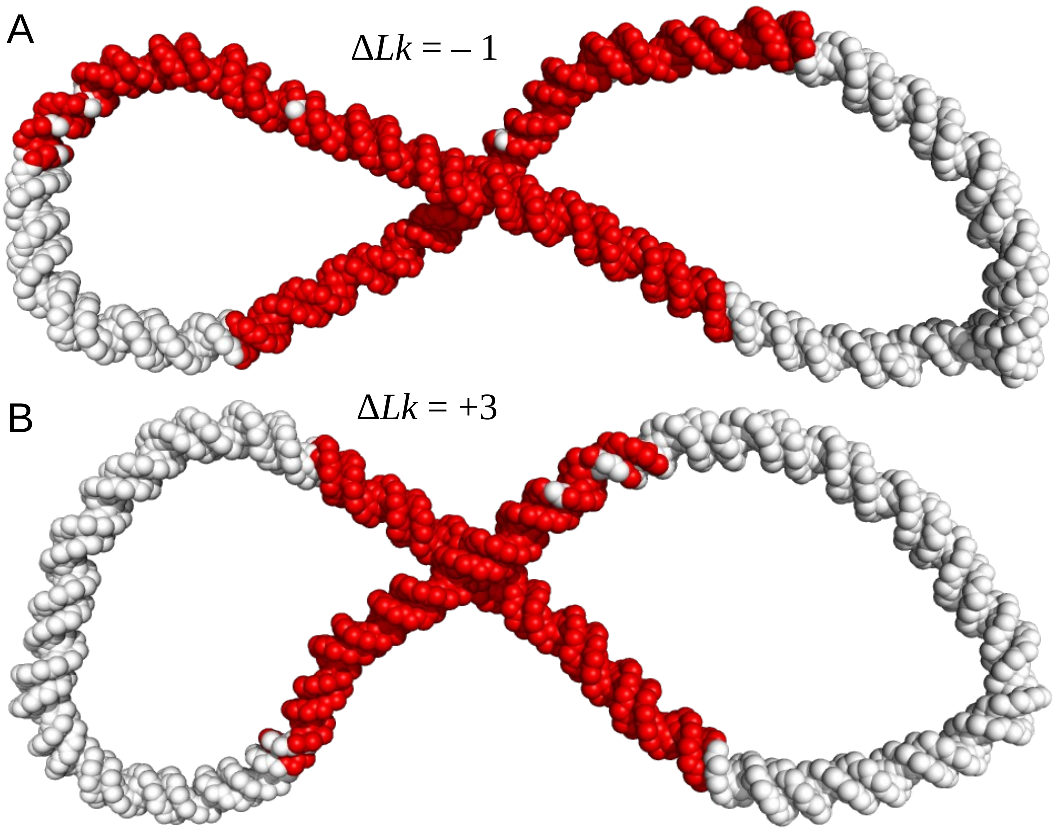

Supplementary Figure S13: The visited DNA sites (marked as red color) by the Fis protein on supercoiled DNA with (A) $\Delta Lk = -1$ and (B) $\Delta Lk = +3$ in a single simulation trajectory. The result clearly suggest that Fis covers more number of DNA sites in $\Delta Lk = -1$ compared to $\Delta Lk = +3$, both having same number of juxtaposition site.

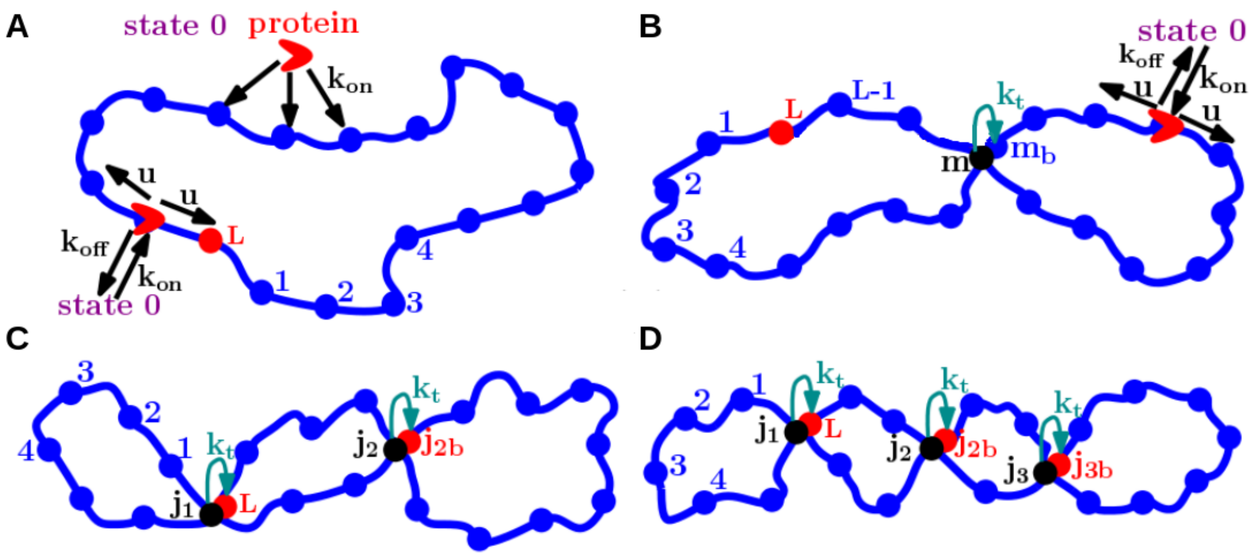

Supplementary Figure S14: General scheme of the discrete-state stochastic approach for the protein target search on supercoiled DNA with (A) no, (B) one, (C) two, and (D) three juxtaposition sites. The searching protein can slide along the DNA with rate u in both the directions or it can diffuse into the solution with rate k_{off}. From the solution, protein can associate to any site on DNA with total rate k_{on}. For supercoiled DNA with juxtaposition sites, the transition rate for intersegmental jumps between two distant DNA sites is k_t. Black, red, and blue circles correspond to the juxtaposition sites, target sites and nonspecific binding sites respectively on DNA of length L.

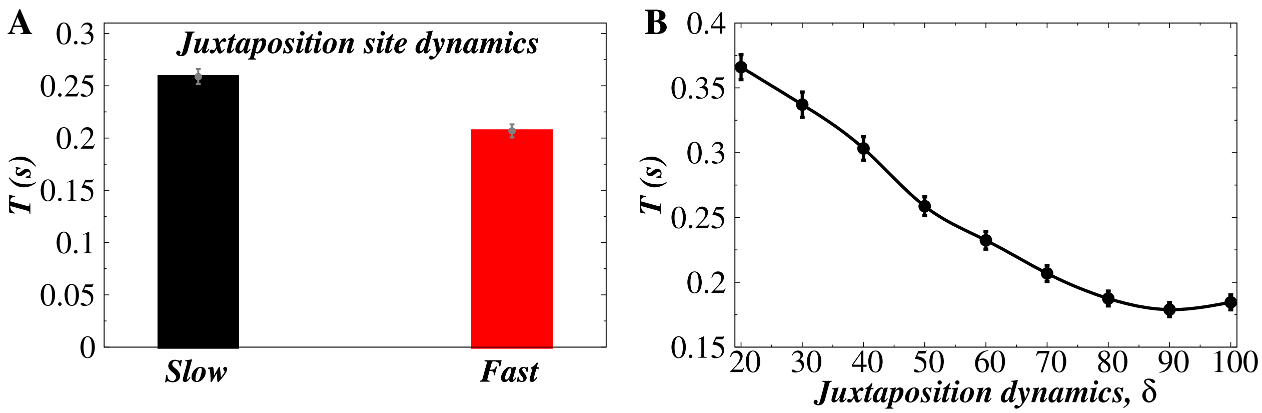

Supplementary Figure S15: (A) Average target search time calculated from Monte Carlo (MC) simulations for a slow ($\delta = 50$) and fast ($\delta = 70$) juxtaposition dynamics on a supercoiled DNA with one juxtaposition site, where δ is defined in Eq. S58. (B) Average target search time as a function of juxtaposition dynamics δ obtained from MC simulations. The target site is positioned at the site L and the juxtaposition site is placed at the site $L/2$. The parameters used for the simulations are $L = 336$ bp, $k_{off} = 9 \times 10^{-5}\ s^{-1}$, $u = 10^3\ s^{-1}$, $k_{on} = 112500\ s^{-1}$ and $k_t = 10^6\ s^{-1}$. The dissociation rate k_{off} of Fis protein is adopted from single-molecule experiment (15) and the association rate k_{on} is obtained from the experimentally measured dissociation constant $K_D = \frac{k_{off}}{k_{on}} = 0.8\ nM$ (16).

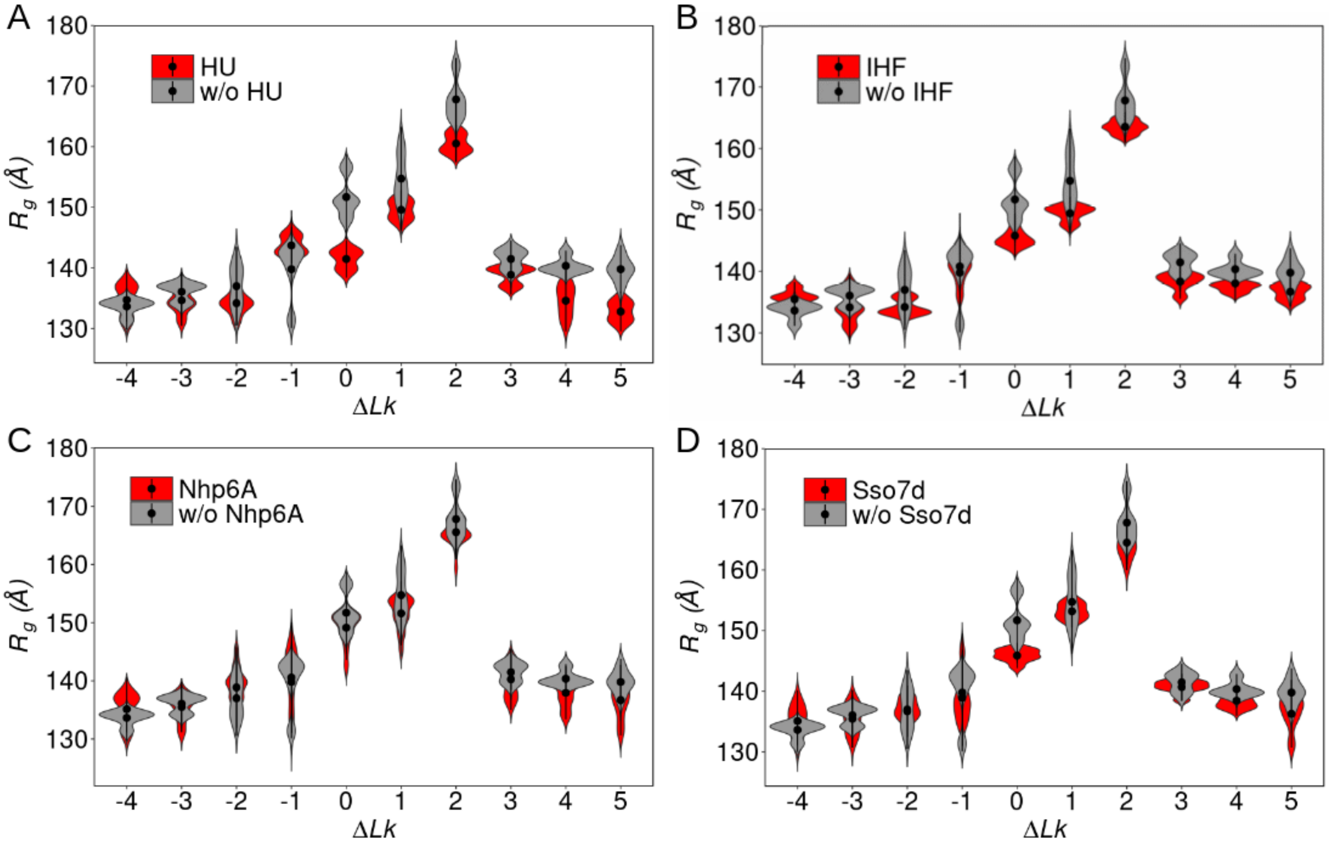

Supplementary Figure S16: The radius of gyration (R_g) of supercoiled DNA in the presence (red) and absence (grey) of (A) HU, (B) IHF, (C) Nhp6A and (D) Sso7d protein are shown as a violin plot for each ΔLk value.

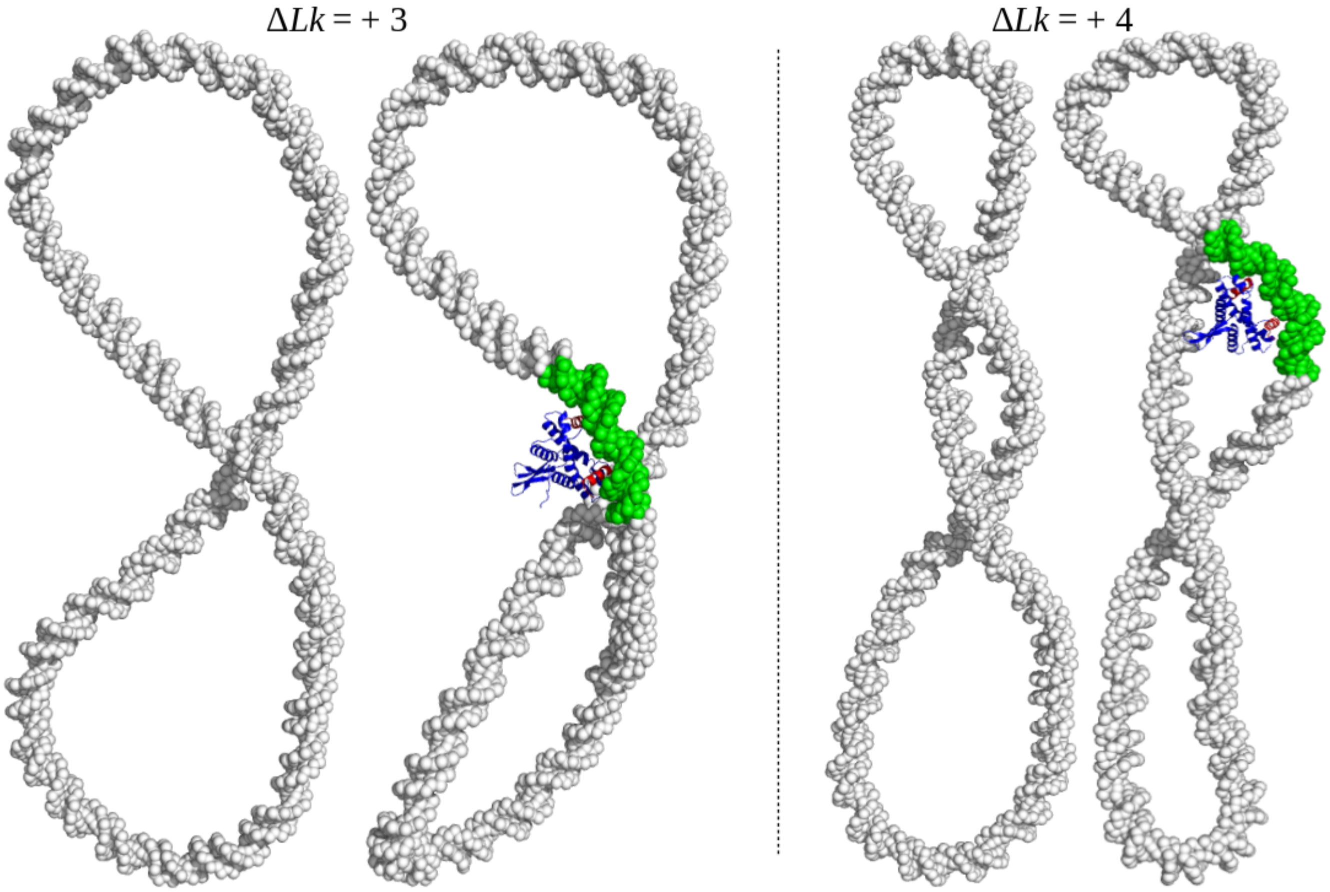

Supplementary Figure S17: Snapshots of supercoiled DNA without and with Fis for $\Delta Lk = +3$ and $\Delta Lk = +4$. The DNA portion colored in green highlights the local DNA bending caused upon binding of Fis protein.

Supplementary Video Legends

Supplementary Video S1: Conversion of minicircle DNA to supercoiled DNA. The conversion of minicircle DNA to Supercoiled DNA with $\Delta Lk = +3$.

Supplementary Video S2: Diffusion of Fis on negatively supercoiled DNA. The diffusivity of Fis protein on supercoiled DNA with $\Delta Lk = -1$. Fis protein is colored white with its recognition region in red color. Double-stranded DNA backbone traces are colored in green and bases are colored in blue.

Supplementary Video S3: Diffusion of Fis on positively supercoiled DNA. The diffusivity of Fis protein on supercoiled DNA with $\Delta Lk = +3$. Fis protein is colored white with its recognition region in red color. Double-stranded DNA backbone traces are colored in green and bases are colored in blue.

References

1. Clementi, C., Nymeyer, H., and Onuchic, J. N. (2000) Topological and energetic factors: what determines the structural details of the transition state ensemble and “en-route” intermediates for protein folding? An investigation for small globular proteins. *J Mol Biol,* **298**, 937–953.

2. Freeman, G. S., Hinckley, D. M., Lequieu, J. P., Whitmer, J. K., and de Pablo, J. J. (2014) Coarse-grained modeling of DNA curvature. *J Chem Phys,* **141**, 165103.

3. Hinckley, D. M., Freeman, G. S., Whitmer, J. K., and de Pablo, J. J. (2013) An experimentally-informed coarse-grained 3-Site-Per-Nucleotide model of DNA: structure, thermodynamics, and dynamics of hybridization. *J Chem Phys,* **139**, 144903.

4. Lequieu, J., Córdoba, A., Schwartz, D. C., and de Pablo, J. J. (2016) Tension-dependent free energies of nucleosome unwrapping. *ACS Cent Sci,* **2**, 660–666.

5. Wang, Q., Irobalieva, R. N., Chiu, W., Schmid, M. F., Fogg, J. M., Zechiedrich, L., and Pettitt, B. M. (2017) Influence of DNA sequence on the structure of minicircles under torsional stress. *Nucleic Acids Res,* **45**, 7633–7642.

6. Irobalieva, R. N., Fogg, J. M., Catanese, D. J., Catanese, D. J., Sutthibutpong, T., Chen, M., Barker, A. K., Ludtke, S. J., Harris, S. A., Schmid, M. F., Chiu, W., and Zechiedrich, L. (2015) Structural diversity of supercoiled DNA. *Nat Commun,* **6**, 8440.

7. Tsai, M. Y., Zhang, B., Zheng, W., and Wolynes, P. G. (2016) Molecular mechanism of facilitated dissociation of Fis protein from DNA. *J Am Chem Soc,* **138**, 13497–13500.

8. Kamagata, K., Mano, E., Ouchi, K., Kanbayashi, S., and Johnson, R. C. (2018) High free-energy barrier of 1D diffusion along DNA by architectural DNA-binding proteins. *J Mol Biol,* **430**, 655–667.

9. Qian, H., Sheetz, M. P., and Elson, E. L. (1991) Single particle tracking. Analysis of diffusion and flow in two-dimensional systems. *Biophys J,* **60**(4), 910–921.

10. Desai, P. R., Brahmachari, S., Marko, J. F., Das, S., and Neuman, K. C. (2020) Coarse-grained modelling of DNA plectoneme pinning in the presence of base-pair mismatches. *Nucleic Acids Res,* **48**(19), 10713–10725.

11. Yagyu, H., Lee, J. Y., Kim, D. N., and Tabata, O. (2017) Coarse-grained molecular dynamics model of double-stranded DNA for DNA nanostructure design. *J Phys Chem B,* **121**(19), 5033–5039.

12. Mitchell, J. S., Laughton, C. A., and Harris, S. A. (2011) Atomistic simulations reveal bubbles, kinks and wrinkles in supercoiled DNA. *Nucleic Acids Res,* **39**, 3928–3938.

13. Sutthibutpong, T., Harris, S. A., and Noy, A. (2015) Comparison of molecular contours for measuring writhe in atomistic supercoiled DNA. *J Chem Theory Comput,* **11**(6), 2768–2775.

14. Mondal, A. and Bhattacherjee, A. (2017) Understanding the role of DNA topology in target search dynamics of proteins. *J Phys Chem B,* **121**, 9372–9381.

15. Kamar, R. I., Banigan, E. J., Erbas, A., Giuntoli, R. D., Olvera de la Cruz, M., Johnson, R. C., and Marko, J. F. (2017) Facilitated dissociation of transcription factors from single DNA binding sites. *Proc Natl Acad Sci U S A,* **114**, E3251–E3257.

16. Skoko, D., Yoo, D., Bai, H., Schnurr, B., Yan, J., McLeod, S. M., Marko, J. F., and Johnson, R. C. (2006) Mechanism of chromosome compaction and looping by the Escherichia coli nucleoid protein Fis. *J Mol Biol,* **364**, 777–798.

17. Putzel, G. G., Tagliazucchi, M., and Szleifer, I. (2014) Nonmonotonic diffusion of particles among larger attractive crowding spheres. *Phys Rev Lett,* **113**, 138302.
